# Inflammation and pancreatic cancer: molecular and functional interactions between S100A8, S100A9, NT-S100A8 and TGFβ1

**DOI:** 10.1186/1478-811X-12-20

**Published:** 2014-03-26

**Authors:** Daniela Basso, Dania Bozzato, Andrea Padoan, Stefania Moz, Carlo-Federico Zambon, Paola Fogar, Eliana Greco, Michele Scorzeto, Francesca Simonato, Filippo Navaglia, Matteo Fassan, Michela Pelloso, Sirio Dupont, Sergio Pedrazzoli, Ambrogio Fassina, Mario Plebani

**Affiliations:** 1 Department of Laboratory Medicine, University-Hospital of Padova, Via Giustiniani 2, 35128 Padova, Italy; 2 Department of Medicine – DIMED, University of Padova, Padova, Italy; 3 Department of Biomedical Sciences – DSB, University of Padova, Padova, Italy; 4 Department of Molecular Medicine, University of Padova, Padova, Italy; 5 Associazione Wirsung Onlus, Padova, Italy

**Keywords:** Akt, Calcium, Calcium binding proteins, Epithelial to mesenchymal transition, Mass spectrometry (MS), Matrix metalloproteinase (MMP), mTOR, Pancreatic cancer, SMAD transcription factor

## Abstract

**Background:**

In order to gain further insight on the crosstalk between pancreatic cancer (PDAC) and stromal cells, we investigated interactions occurring between TGFβ1 and the inflammatory proteins S100A8, S100A9 and NT-S100A8, a PDAC-associated S100A8 derived peptide, in cell signaling, intracellular calcium (Ca_i_^2+^) and epithelial to mesenchymal transition (EMT). NF-κB, Akt and mTOR pathways, Ca_i_^2+^ and EMT were studied in well (Capan1 and BxPC3) and poorly differentiated (Panc1 and MiaPaCa2) cell lines.

**Results:**

NT-S100A8, one of the low molecular weight N-terminal peptides from S100A8 to be released by PDAC-derived proteases, shared many effects on NF-κB, Akt and mTOR signaling with S100A8, but mainly with TGFβ1. The chief effects of S100A8, S100A9 and NT-S100A8 were to inhibit NF-κB and stimulate mTOR; the molecules inhibited Akt in Smad4-expressing, while stimulated Akt in Smad4 negative cells. By restoring Smad4 expression in BxPC3 and silencing it in MiaPaCa2, S100A8 and NT-S100A8 were shown to inhibit NF-κB and Akt in the presence of an intact TGFβ1 canonical signaling pathway. TGFβ1 counteracted S100A8, S100A9 and NT-S100A8 effects in Smad4 expressing, not in Smad4 negative cells, while it synergized with NT-S100A8 in altering Ca_i_^2+^ and stimulating PDAC cell growth. The effects of TGFβ1 on both EMT (increased Twist and decreased N-Cadherin expression) and Ca_i_^2+^ were antagonized by S100A9, which formed heterodimers with TGFβ1 (MALDI-TOF/MS and co-immuno-precipitation).

**Conclusions:**

The effects of S100A8 and S100A9 on PDAC cell signaling appear to be cell-type and context dependent. NT-S100A8 mimics the effects of TGFβ1 on cell signaling, and the formation of complexes between TGFβ1 with S100A9 appears to be the molecular mechanism underlying the reciprocal antagonism of these molecules on cell signaling, Ca_i_^2+^ and EMT.

## Lay abstract

### Background

S100A8/A9, NT-S100A8 are overexpressed in PDAC stroma when cancer cells express Smad4, suggesting that they are linked with TGFβ1 signalling.

### Results

S100A8 inhibits NF-kB and activates Akt in Smad4+, not in Smad4- cells. NT-S100A8 mimics TGFβ1. S100A9 binds to and antagonizes TGFβ1.

### Conclusion

S100A8 signaling in PDAC cells is Smad4-dependent.

### Significance

Novel interactions between S100 proteins and TGFβ1 signaling in PDAC have been identified.

## Background

Pancreatic ductal adenocarcinoma (PDAC), the fourth leading cause of cancer-related mortality in the U.S.A. and Europe [1,2], is an extremely aggressive form of cancer [3]. When the widely known PDAC precursor, PanIN, progresses from stage I to stage III, it accumulates cellular atypia and a series of genetic alterations, including activation of oncogenic *k-ras* and inactivation of tumor suppressors *p16Ink4a* and *p53*[4]. As clearly demonstrated in genetically engineered mouse models of PDAC progression, the pancreatic epithelium-specific expression of *k-ras* (KrasG12D) induces PanIN lesions that can progress to PDAC, the average latency being more than one year; K-rasG12D expression coupled with the homozygous or heterozygous deletion of the tumor suppressor genes *p53* and/or *p16Ink4a* underlies short latency PDAC [5-7]. In humans and mice, PDAC progression is characterized by the inactivation of the tumor suppressor gene, *Smad4*[4,8]. In most cases of PDAC, *k-ras*, *p16Ink4a* and *p53* mutations are present [8], whereas in about 50% of cases, *Smad4* inactivation due to gene deletion or mutations associated with loss of heterozygosity, is identified [9]. When *Smad4* is inactivated, PDAC is associated with widespread metastases [10] and a poorer prognosis [11].

The transcription factor encoded by *Smad4* is a central effector of the transforming growth factor beta 1 (TGFβ1) signaling pathway [12], which is one of the 12 core signaling pathways reported to be altered in most PDAC cases [13]. TGFβ1 is critically involved in cancer initiation and progression through tumor cell autonomous signaling and interactions with the tumor microenvironment [14]. Coupled with the type II TGFβ receptor, TGFβ1 activates the type I receptor, which then triggers the canonical and/or non-canonical pathways. In the canonical pathway it recruits and phosphorylates receptor Smads (Smad2 and Smad3) thus facilitating their interaction with Smad4 and its subsequent nuclear translocation, while in the non-canonical pathway it activates a series of signaling cascades including Ras, PI3K and NF-κB [12,15]. The effects of TGFβ1 stimulation vary in different settings, since the response to this molecule is cell-type and context dependent. In PDAC, TGFβ1 acts as a tumor suppressor by inhibiting cancer cell growth, and as a tumor promoter by dampening host immune surveillance while inducing an epithelial to mesenchymal transition (EMT) and a stromal expansion [16-19]. EMT confers on tumor cells a capacity to migrate and metastasize [17], while stromal expansion, which is marked in PDAC [20], creates a favorable setting for tumor cell migration and disease progression [21,22].

The TGFβ1 molecule is a crucial component in understanding tumor-stromal interactions: although produced by tumor cells, it is overexpressed by stromal cells mainly at the invasive front area of human PDAC [23]; moreover, in an experimental PDAC mouse model TGFβ1 was shown to mediate cancer cell derived Cxcr2 stimulatory effects on pancreatic fibroblasts to express Ctgf, which is greatly involved in PDAC progression [21].

In investigating tumor-stromal interactions, particular attention should be paid to the inflammatory proteins S100A8 and S100A9 [24], which are encoded by genes clustered on chromosome 1q21 [25]. This chromosome region [26,27] as well as S100A8/A9 TLR4 and RAGE receptors [28-30], has been associated with cancer development and metastasis. In PDAC, S100A8/A9 are overexpressed by infiltrating inflammatory cells [31,32], the levels of expression appearing to be correlated with TGFβ1 signaling: *Smad4* depletion is associated with reduced S100A8 (not S100A9) positive infiltrating inflammatory cells [31,33] and, conversely, these proteins are expressed by PDAC cell lines only in cases of *Smad4* inactivation [34]. The existence of an interaction between S100A8, S100A9 and the TGFβ1 canonical signaling pathway is supported by the finding that both molecules induce Smad1 and Smad2 phosphorylation through a RAGE dependent mechanism [33]. Since S100A8 and S100A9 are calcium binding proteins, the complexity of their effects on tumor biology may depend not only on TLR4 and/or RAGE ligation, but also on alterations in intracellular calcium fluxes and/or concentrations, demonstrated to occur in pancreatic β-cells exposed to the 14 N-terminal aminoacid peptide of S100A8 (NT-S100A8), which we isolated from PDAC tissues of patients with PDAC-associated diabetes mellitus [34]. Variations in intracellular calcium, critical events that regulate cancer cell proliferation and progression, may be explained by the complex effects of calcium on different cancer cell signaling pathways, including the Ca-calmodulin protein kinase cascade, Akt, mTOR and TGFβ1 [35-40]. It is not known whether the release of NT-S100A8 from the entire S100A8 is a PDAC-driven event and whether these two molecules, and the S100A8 binding partner S100A9, interact with TGFβ1 in altering PDAC signaling pathways and cell biology.

The end points of the present study were therefore to ascertain whether: 1. PDAC cells express S100A8/A9 proteins and PDAC-derived proteases fragment S100A8/A9 causing the release of their N-terminal peptides, with a special focus on NT-S100A8; 2. TGFβ1 and/or an intact TGFβ1 signaling pathway influence the S100A8/A9 and NT-S100A8 effects on Akt, NF-κB and mTOR signaling pathways in PDAC cells; 3. S100A8/A9 and NT-S100A8 affect intracellular calcium fluxes in a TGFβ1 dependent or independent manner; 4. S100A8/A9 and NT-S100A8 modify PDAC cell proliferation, apoptosis and the EMT process directly and/or by interfering with TGFβ1 activity.

In the present study we demonstrate that NT-S100A8 and S100A8 preferentially activate mTOR in cells with wild type Smad4 expression and NF-κB in Smad4 negative cells, and that, by forming complexes, S100A8/A9 and TGFβ1 antagonize the effects of each other on intracellular calcium and EMT.

## Results

### Metastatic PDAC cell lines have high S100A8/A9 expression

A comparative quantification was made of S100A8 and S100A9 mRNA expression by real time PCR and referred to their respective expression levels by the PDAC metastatic SUIT2 cell line (Table 1). Capan1 expressed high S100A8 and S100A9 mRNA levels. MiaPaCa2 and Panc1 did not express detectable S100A8 mRNA levels, and had a S100A9 expression 100 fold lower than SUIT2. BxPC3 did not express detectable levels of either S100A8 or S100A9 mRNA, while the stable transfected BxPC3-SMAD4+ cell line (BxPC3-SMAD4+) had S100A9 (not S100A8) expression comparable to that of MiaPaCa2 and Panc1 (100 fold lower than SUIT2). S100A8/A9 receptor RAGE expression, found in all studied cell lines, was lower in BxPC3 and Panc1 than in Capan1 and MiaPaCa2 (Additional file 1: Figure S1).

**Table 1:** S100A8 and S100A9 mRNA relative quantification in PDAC cell lines

**PDAC cell lines**	**S100A8 fold increase**	**S100A9 fold increase**	**Reference (SUIT2)**
	**Mean ± SD**	**Mean ± SD**	
**BxPC3**	Absent	Absent	1
**Capan1**	1736 ± 230	582.92 ± 18.83	1
**MiaPaCa2**	Absent	0.01 ± 0.000798	1
**Panc1**	Absent	0.01 ± 0.000997	1
**BxPC3-Smad4+**	Absent	0.01 ± 0.003447	1

The PDAC metastatic SUIT2 cell line was used as reference (S100A8 C_T_ = 36.46 ± 0.34 cycles; S100A9 C_T_ = 32.01 ± 0.53 cycles; mean ± SD from three independent experiments, each made in duplicate).

### S100A8 proteolysis is induced by pancreatic cancer cell conditioned media

**Figure 1:**
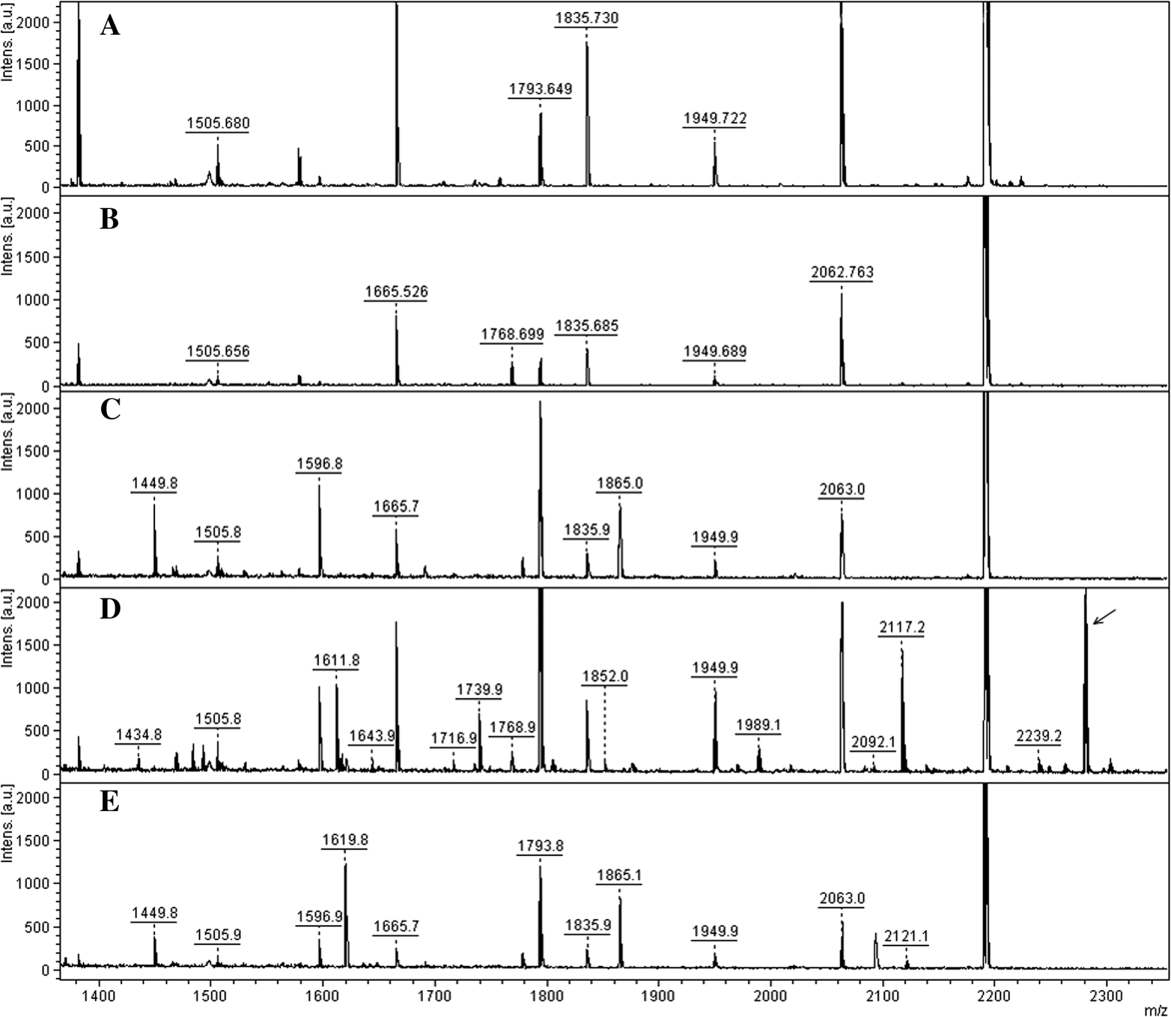
**S100A8 is fragmented by PDAC cell conditioned media.** MALDI-TOF/MS low-molecular weight spectra obtained after overnight incubation of S100A8 in complete control culture medium, in Capan1 conditioned media (CM) and in Ukrain treated Capan1 CM. **A:** complete control medium; **B:** complete control medium added with 3 μM S100A8; **C:** Capan1 conditioned medium; **D:** Capan1 conditioned medium with added 3 μM S100A8; **E:** Ukrain treated Capan1 conditioned medium with added 3 μM S100A8. The complete control medium was incubated at 37°C in a cell culture incubator without cells for 96 hours with 1% FCS and 0.1% gentamycin, in the same conditions as CM. The arrow indicates the 2280 m/z peptide.

**Figure 2:**
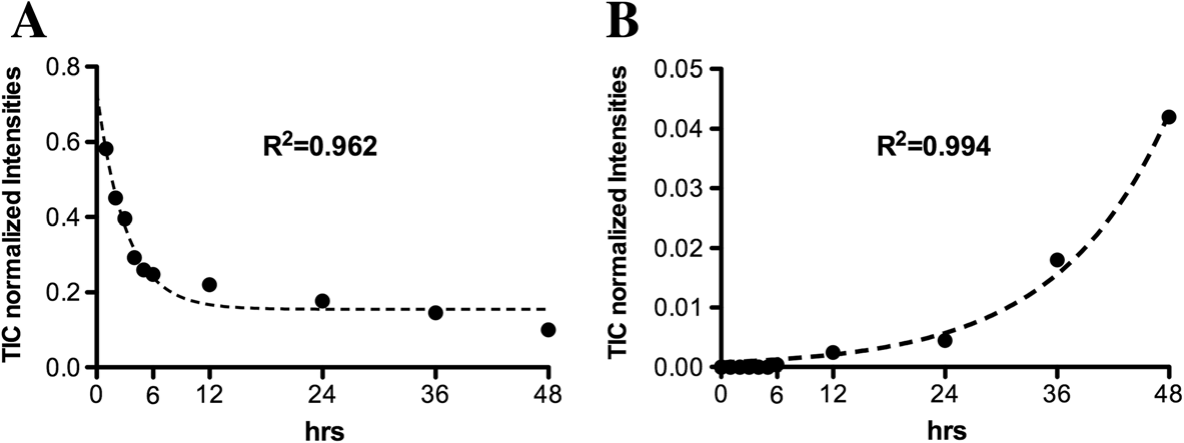
**Degradation kinetics of S100A8 by PDAC cell conditioned media.** S100A8 (3 μM) was incubated with Capan1 conditioned media for 1, 2, 3, 4, 5, 6, 12, 24, 36 and 48 hours at 37°C before MALDI-TOF/MS analyses. The intensities of S100A8 and of the main degradation peptide identified (2280 m/z) were normalized to total ion current (TIC). **A:** S100A8 TIC normalized intensities; **B:** 2280 m/z peptide TIC normalized intensities. The curve kinetics were obtained by exponential fitting.

**Figure 3:**
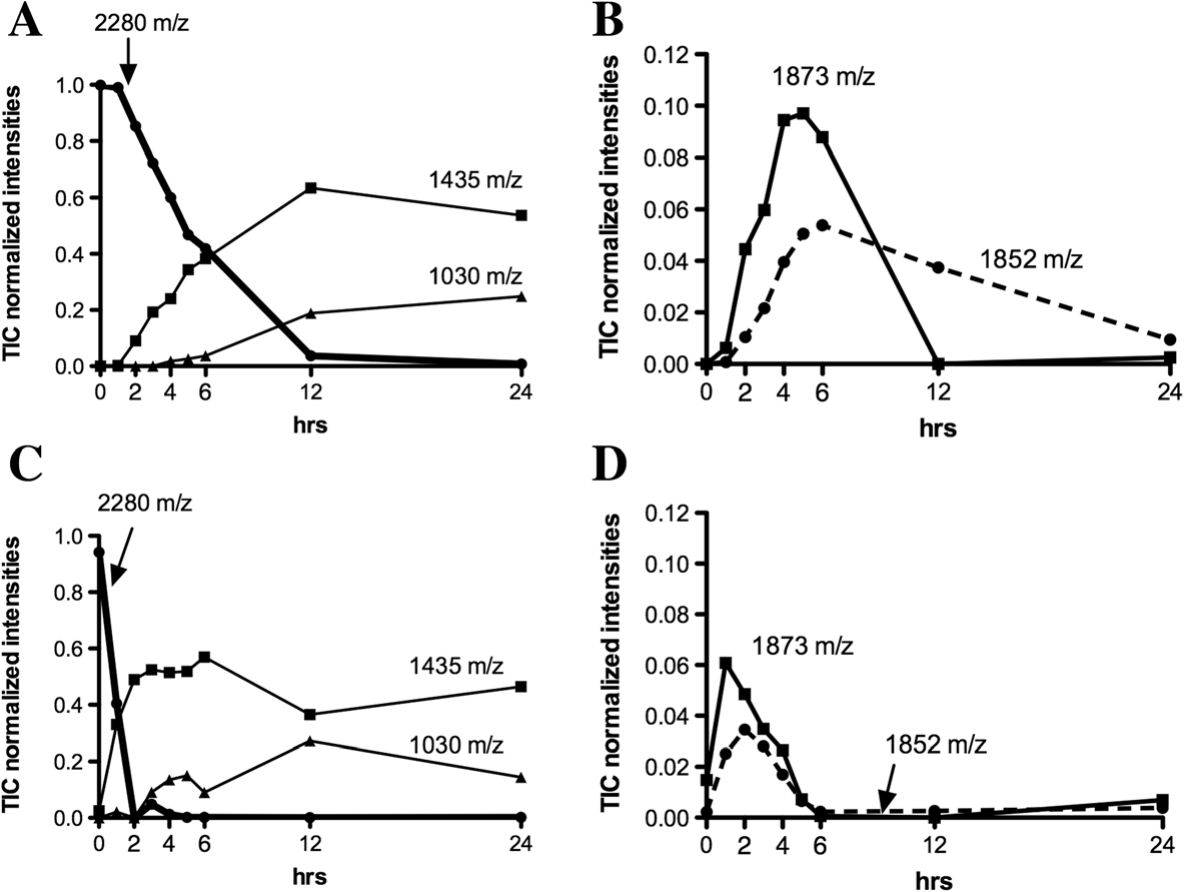
**Degradation kinetics of the 2280 m/z peptide by PDAC cell conditioned media.** The 2280 m/z peptide (200 μM), encompassing the 1–19 aminoacid sequence of S100A8, was incubated with BxPC3 (**A** and **B**) or Capan1 (**C** and **D**) conditioned media for 1, 2, 3, 4, 5, 6, 12 and 24 hours at 37°C before MALDI-TOF/MS analyses. The intensities of the main five peptides identified were normalized to total ion current (TIC). In non-conditioned media the abundance of the parental 19 aminoacid peptide at 2280 m/z was 0.99 both at time 0 and after 24 hours.

Figure 1 shows low molecular weight MALDI-TOF/MS spectra obtained after 24 hours’ incubation at 37°C of S100A8 in complete control culture medium, Capan1 conditioned medium (CM) and Capan1 CM obtained after treatment with the MMPs inhibitor Ukrain [41]. On incubating S100A8 with Capan1 CM, a new peptide was found at 2280 m/z (Figure 1D). This peptide progressively accumulated as the incubation time of S100A8 in Capan1 CM increased, the kinetic of its appearance being inverse with respect to that of S100A8 degradation (Figure 2). The 2280 m/z peptide was not detected when S100A8 was incubated in Ukrain treated Capan1 CM (Figure 1E). At MALDI-TOF-MS/MS analysis using de-novo sequencing, the 2280 m/z peptide was identified as the 1–19 N-terminal aminoacid sequence of S100A8 (Additional file 2: Figure S2). The 1–19 N-terminal aminoacid sequence of S100A8 was then synthesized and incubated for 1, 2, 3, 4, 5, 6, 12 and 24 hours in the control, Capan1 and BxPC3 CM at 37°C. At the end of all incubation times, MALDI-TOF/MS analysis was made in the low molecular weight range (650–3000 m/z). In the presence of both BxPC3 and Capan1 CM, after 24 hours’ incubation the parental 19 aminoacid peptide disappeared, while a series of new peptides were detected, the most abundant being those at 1030, 1252, 1435, 1852, 1873 m/z; two of these, at 1435 and 1852 m/z, were also detected in the spectra from S100A8 incubated with Capan1 CM (Figure 1D). Using the proteomic tool, FindPept, the above listed peptides were identified as the 2–10, 10–19, 8–19, 1–16 and 3–18 aminoacid sequences of S100A8, respectively. At MALDI-TOF-MS/MS analysis of the 1435 m/z peptide, its correspondence to the 8–19 S100A8 aminoacid sequence was confirmed (Additional file 3: Figure S3). Figure 3 shows the variations over time in the abundance of 1030 and 1435 m/z, and of the 1852 and 1873 m/z peptides in BxPC3 and Capan1 CM.

### Effects and interactions of S100 proteins and TGFβ1 on NF-κB, Akt, mTOR and STAT signaling in PDAC cells

**Figure 4:**
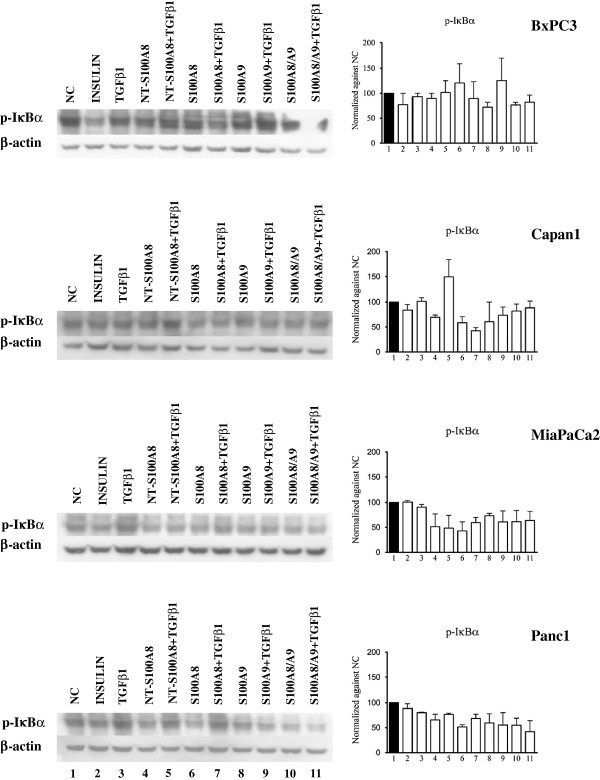
**Differential effects of S100 proteins and TGFβ1 on NF-κB signaling.** BxPC3, Capan1, MiaPaCa2 and Panc1 remained unstimulated (NC, negative control) or were stimulated for 10 min with 50 mU insulin (positive control), 0.02 ng/ml TGFβ1 alone or combined with 10 nM S100A8, 10 nM S100A9, 10 nM S100A8/A9 complex, 50 nM NT-S100A8. Western blot show IκBα phosphorylation at Ser^32^ site and the corresponding β-actin, used as the control. Histograms show semi-quantification of band intensities after normalization against the negative control (O.D.; ImageJ software, v 1.47). Columns indicate mean values, bars indicate SD from two independ experiments.

To obtain a representative picture “in vitro” of the complexity of human PDAC, our study included the four pancreatic cancer cell lines BxPC3, Capan1, MiaPaCa2 and Panc1, which differ in their genetic make-up, grade and metastatic potential [42]. Using these cell lines the effects of S100A8, S100A9 and of NT-S100A8, alone or combined with TGFβ1, on NF-κB, Akt, mTOR and STAT signaling pathways were evaluated. The response to insulin stimulation was also analyzed as a reference for the PI3K pathway. To investigate NF-κB signaling, we analyzed IκBα phosphorylation (Figure 4) that, when enhanced, is associated with IκBα ubiquitination and the nuclear translocation of NF-κB dimers, which activate the transcription of their target genes.

**Figure 5:**
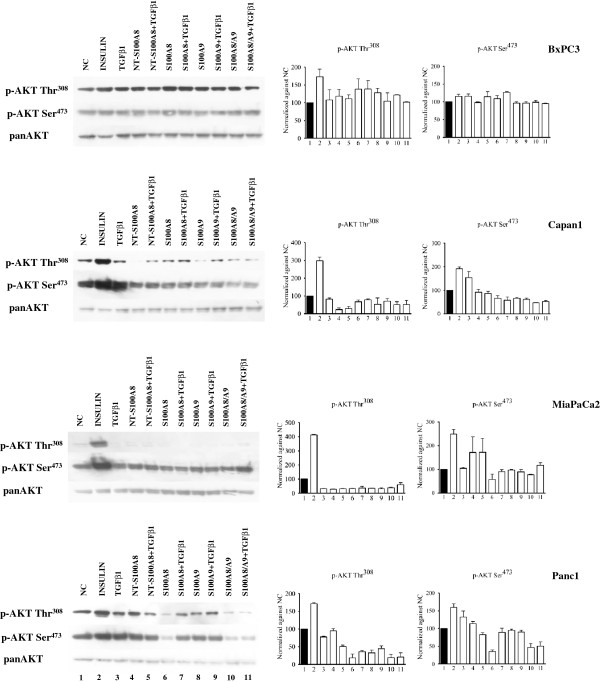
**Differential effects of S100 proteins and TGFβ1 on Akt signaling.** BxPC3, Capan1, MiaPaCa2 and Panc1 remained unstimulated (NC, negative control) or were stimulated for 10 min with 50 mU insulin (positive control), 0.02 ng/ml TGFβ1 alone or combined with 50 nM NT-S100A8, 10 nM S100A8, 10 nM S100A9, 10 nM S100A8/A9 complex. Western blot show the Akt phosphorylation sites Thr^308^ and Ser^473^ and the corresponding non-phosphorylated Akt (panAkt), used as the control. Histograms show semi-quantification of band intensities after normalization against the negative control (O.D.; ImageJ software, v 1.47). Columns indicate mean values, bars indicate SD from two independ experiments.

**Figure 6:**
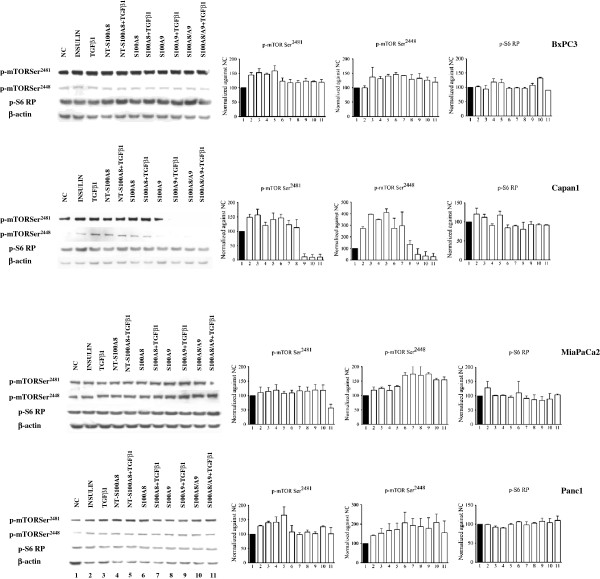
**Differential effects of S100 proteins and TGFβ1 on mTOR signaling.** BxPC3, Capan1, MiaPaCa2 and Panc1 remained unstimulated (NC, negative control) or were stimulated for 10 min with 50 mU insulin (positive control), 0.02 ng/ml TGFβ1 alone or combined with 50 nM NT-S100A8, 10 nM S100A8, 10 nM S100A9, 10 nM S100A8/A9 complex. Western blot showed the mTOR phosphorylation sites Ser^2481^ and Ser^2448^, the corresponding phosphorylation of S6 Ribosomal Protein at the Ser^235/236^ site (pS6RP) and β-actin, used as the control. Histograms show semi-quantification of band intensities after normalization against the negative control (O.D.; ImageJ software, v 1.47). Columns indicate mean values, bars indicate SD from two independ experiments.

S100A8, S100A9, S100A8/A9 and NT-S100A8 exerted overlapping effects on NF-κB signaling: they reduced IκBα phosphorylation in Capan1, MiaPaCa2 and Panc1, not in BxPC3. TGFβ1 had no significant effects on IκBα phosphorylation. We then studied the Akt signaling pathway by western blot analysis of Thr^308^, induced by the PI3K, and Ser^473^, a target site of mTORC2 [43] (Figure 5). S100A8, S100A9 and S100A8/A9 uniformly induced in BxPC3, and reduced in the other cell lines, Akt Thr^308^ phosphorylation. These molecules, but mainly S100A8 caused also a reduced Akt Ser^473^ phosphorylation in Capan1, MiaPaCa2 and Panc1 cells. NT-S100A8, similarly to S100A8, reduced in Capan1 and MiaPaCa2 Thr^308^ phosphorylation, but unlike S100A8 this peptide augmented in the same cell lines Ser^473^. TGFβ1 co-treatment caused no relevant modification in findings for the effects of S100 calcium binding proteins on Akt phosphorylation, with the exception of S100A8 and NT-S100A8 effects in Panc1 cells. mTOR signaling was then studied by analyzing mTOR Ser^2448^, mTOR Ser^2481^ and S6 Ribosomal Protein (S6RP) phosphorylation (Figure 6). S100A8 and S100A9 induced mTOR Ser^2448^ phosphorylation in all cell lines and Ser^2481^ in BxPC3 and Capan1; S100A8/A9 induced in BxPC3, MiaPaCa2 and Panc1, but reduced in Capan1, mTOR Ser^2481^ and Ser^2448^ phosphorylation and induced S6RP phosphorylation in BxPC3. NT-S100A8 and TGFβ1 had shared effects on mTOR, both molecules inducing Ser^2481^ and Ser^2448^ phosphorylation in all cell lines. In Capan1 S100A9 and TGFβ1 co-treatment abolished mTOR Ser^2481^ and Ser^2448^ phosphorylation induced by any of these molecules. STAT3, constitutively phosphorylated in all studied PDAC cell lines, was minimally affected by S100A8/A9 and TGFβ1 co-treatment only, which reduced in MiaPaCa2 and induced in Panc1 Stat3 phosphorylation (Additional file 4: Figure S4).

Overall the above data indicate that the S100A8, S100A9 and S100A8/A9 heterocomplex trigger the same signaling pathways, although any molecule may evoke independent effects depending on the PDAC cell type. S100A8, S100A9 and S100A8/A9 inhibit NF-κB and Akt in PDAC cells with an intact Smad4, but not in cells (BxPC3) bearing Smad4 homozygous deletion. The independent actions of S100A8/A9 heterocomplex and of S100A9 were particularly evident in Capan1 and Panc1 cells: like S100A8 and S100A9, S100A8/A9 inhibits NF-κB, but unlike these molecules rather than stimulating it inhibits mTOR in Capan1; conversely S100A9, unlike S100A8 and S100A8/A9, was less inhibitory on Akt in Panc1. Intriguingly, NT-S100A8 and TGFβ1 have shared effects on Akt and mTOR and, as reported for S100 proteins, their effects are cell type dependent: both molecules inhibited Akt Thr^308^, but not Akt Ser^473^ phosphorylation in Smad4 expressing cells (Capan1, MiaPaCa2 and Panc1) and activated mTOR in all cell lines. Finally, TGFβ1 antagonizes the effects of S100A8 on Akt Ser^473^ in MiaPaCa2 and Panc1 and those of S100A9 on mTOR in Capan1, but none of the NT-S100A8 effects.

### Smad4 deletion drives the NF-κB and Akt and reduces the mTORC1 response to S100A8

**Figure 7:**
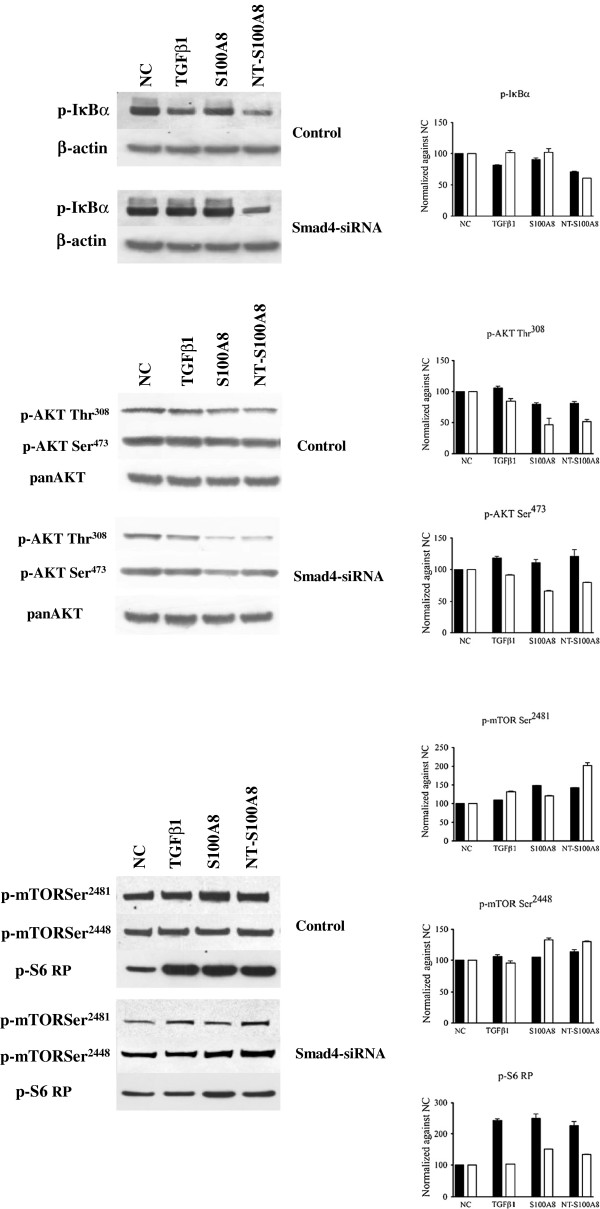
**Effects of Smad4 deletion on NF-κB, Akt and mTOR signaling.** MiaPaCa2 (Control) and Smad4 silenced MiaPaCa2 (Smad4-siRNA) remained unstimulated (NC, negative control) or were stimulated for 10 minutes with 0.02 ng/ml TGFβ1, 10 nM S100A8 or 50 nM NT-S100A8. Upper panels show IκBα phosphorylation at Ser^32^ site and corresponding β-actin, used as the control. Middle panels show Akt phosphorylation sites Thr^308^ and Ser^473^ and the corresponding non-phosphorylated Akt, used as the control. Lower panels show mTOR phosphorylation sites Ser^2481^ and Ser^2448^, and the S6 Ribosomal Protein phosphorylation at Ser^235/236^ site. Histograms show semi-quantification of band intensities after normalization against the negative control (O.D.; ImageJ software, v 1.47). Columns indicate mean values, bars indicate SD from two independ experiments. Black colomuns: MiaPaCa2 cells; white columns: Smad4-siRNA MiaPaCa2 cells.

In order to focus on Smad4 as a potential regulator of the different effects induced by the same molecule in different cell types, we studied BxPC3, which do not express Smad4, and the stable transfected BxPC3-SMAD4+ cell line. These two cell lines were treated with all the studied molecules in the above-described conditions, and NF-κB, Akt, mTOR and STAT signaling were then analyzed (Additional file 5: Figure S5). Smad4 expression caused a reduced IκBα phosphorylation after S100A8 treatment, and a reduced Akt Thr^308^ phosphorylation after S100A8/A9 treatment; these effects were not reverted by TGFβ1. When Smad4 expression was restored in BxPC3, it counteracted S100A8/A9, but not S100A8, S100A9, NT-S100A8 or TGFβ1 induced mTOR Ser^2481^ phosphorylation. To further verify whether Smad4 impacted on the effects of S100A8, NT-S100A8 and TGFβ1 on NF-κB, Akt and mTOR signaling, we analyzed IκBα, Akt, mTOR and S6RP phosphorylation in MiaPaCa2, which are Smad4 expressing cells, and in Smad4 silenced MiaPaCa2 (Figure 7). In agreement with the hypothesis deriving from BxPC3 and BxPC3-SMAD4+ findings that Smad4 expression permits NF-κB inhibition, we found that Smad4 silencing reduced the inhibition of NF-κB after TGFβ1 and S100A8, but not after NT-S100A8 stimulation. Moreover, silenced Smad4 markedly reduced S6RP phosphorylation following exposure to TGFβ1, but also to S100A8 or NT-S100A8 treatment which, in Smad4 silenced cells, also induced mTOR, and reduced Akt Thr^308^ and Ser^473^ phosphorylation.

The above data indicate that Smad4 is critically involved not only in TGFβ1, but also in S100A8 and NT-S100A8 signaling: these molecules preferentially activate mTOR in wild type Smad4 expressing cells, while in Smad4 negative cells S100A8 preferentially activates NF-κB and mTOR while inhibiting Akt signaling.

### The effects and interactions of TGFβ1 and S100 proteins in pancreatic cancer cell calcium flows

**Figure 8:**
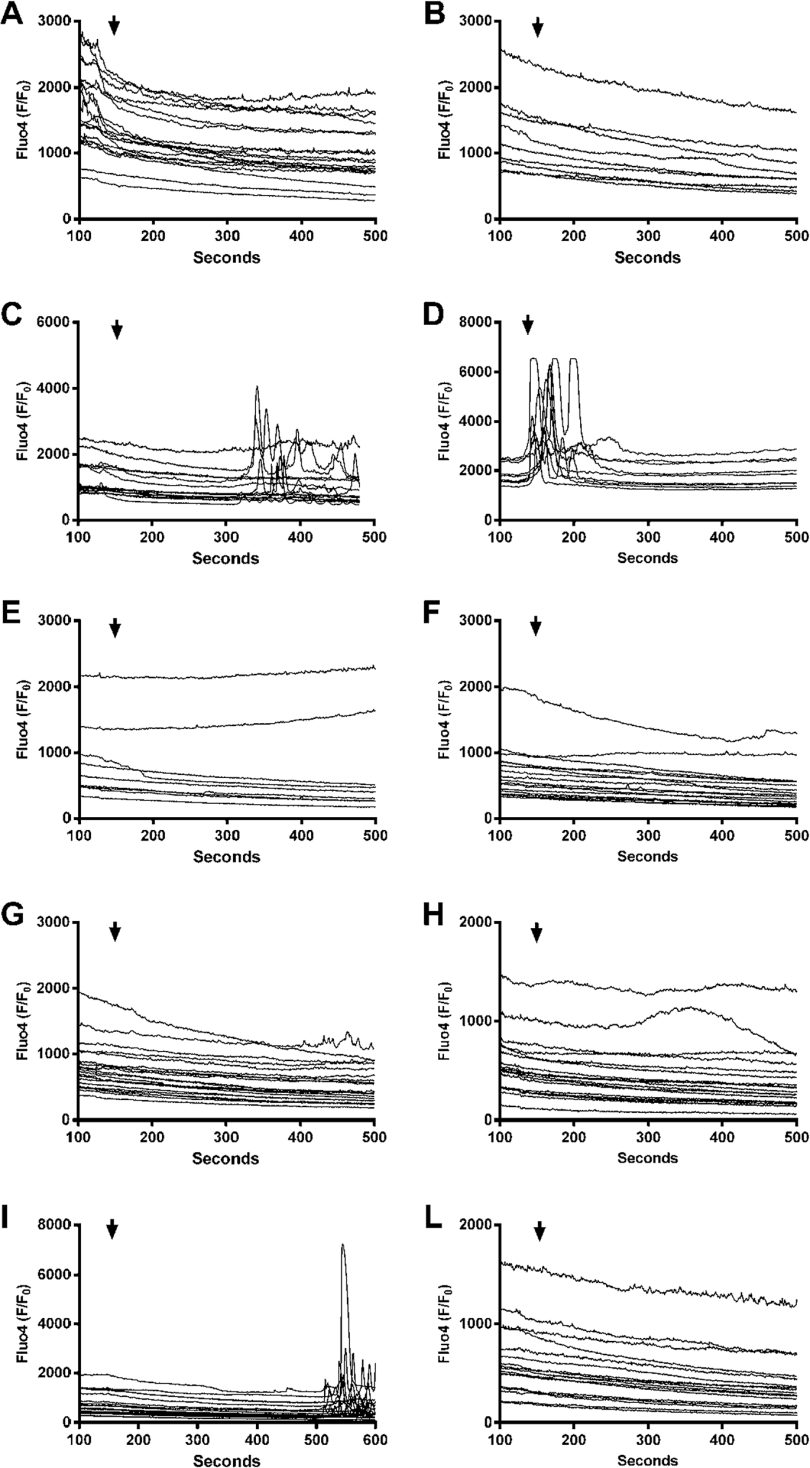
**Intracellular calcium concentration ([Ca**^**2+**^**]**_**i **_**) in BxPC3 cells.** [Ca^2+^]_i_ was monitored for 500 seconds by epifluorescence after stimuli (arrows). Each of the multiple lines in the graphs represent the [Ca^2+^]_i_ of one cell. **A:** Control; **B:** TGFβ1; **C:** NT-S100A8; **D:** NT-S100A8 plus TGFβ1; **E:** S100A8; **F:** S100A8 plus TGFβ1; **G:** S100A9; **H:** S100A9 plus TGFβ1; **I:** S100A8/A9; **L:** S100A8/A9 plus TGFβ1.

**Figure 9:**
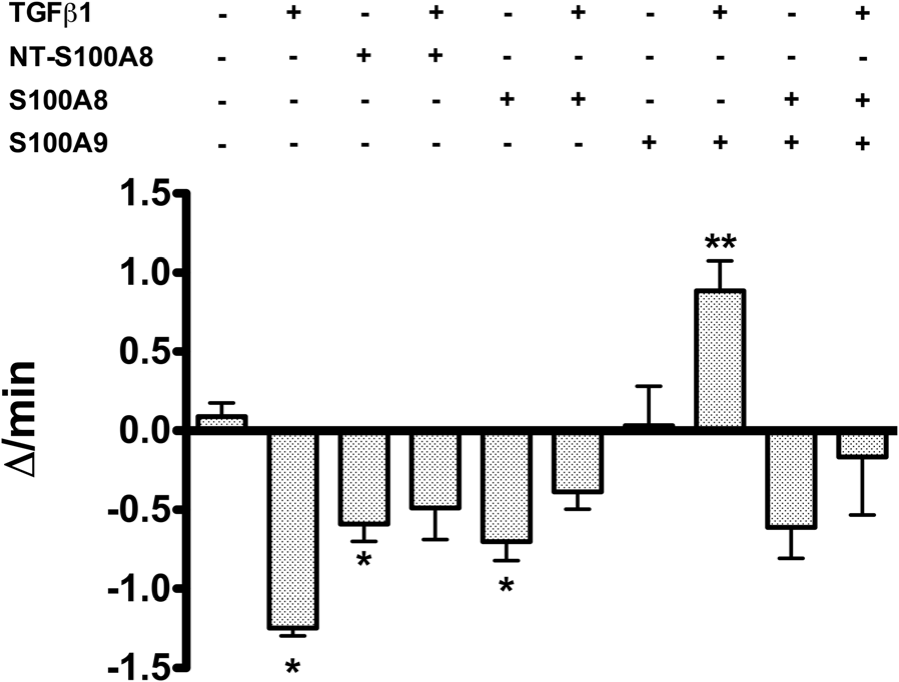
**Intracellular calcium ([Ca**^**2+**^**]**_**i **_**) variations in MiaPaCa2 cells.** Increment (from 0 to 1.5 y-axis) or decrement (from 0 to −1.5 y-axis) in [Ca^2+^]_i_ per min per cell were calculated within a 200 to 480 second interval in the different experimental sets. Columns represent mean values, and bars, standard errors. Bonferroni’s test for pairwise comparisons: * = p < 0.05 with respect to the unstimulated cells; ** = p < 0.05 with respect to unstimulated and p < 0.01 with respect to TGFβ1 stimulated cells.

In order to ascertain whether TGFβ1 and S100 proteins affect intracellular calcium, a key ion involved in the regulation of multiple signaling pathways, the intracellular calcium concentration ([Ca^2+^]_i_) was monitored for 500 seconds while Flou4 fluorescence intensity was measured in two representative PDAC cell lines, BxPC3 and MiaPaCa2. Figure 8 shows the BxPC3 data; each of the multiple lines in the graphs represent the [Ca^2+^]_i_ of one cell. Single treatment with TGFβ1, S100A8 or S100A9 did not determine evident alterations in [Ca^2+^]_i_, while the S100A8/A9 complex caused a cascade of [Ca^2+^]_i_ oscillations after 400 seconds (Figure 8I); this effect was counteracted by TGFβ1 (Figure 8L). NT-S100A8 induced [Ca^2+^]_i_ oscillations after 300 seconds (Figure 8C); this effect was synergized and accelerated by TGFβ1 (Figure 8D). No variation in [Ca^2+^]_i_ was observed in the different experimental conditions investigated in MiaPaCa2. However, since an increasing or decreasing trend in [Ca^2+^]_i_ was observed in the different experimental sets, we calculated the increment or decrement in [Ca^2+^]_i_ per minute per cell within a 200 to 480 second interval. Figure 9 reports mean values and standard errors of the percentage variations obtained in the different experimental sets (One-Way ANOVA: F = 12.17, p < 0.0001). TGFβ1, S100A8 and NT-S100A8 caused a significant decrement of [Ca^2+^]_i_ with respect to control cells. The decrement induced by TGFβ1 was partly counteracted by S100A8 and S100A8/A9, and completely reverted by S100A9. These data indicate that intracellular calcium is altered by the S100A8, S100A9, S100A8/A9 heterocomplex and by NT-S100A8, these alterations being molecule- and cell type-dependent. The effects of S100A8/A9 are antagonized by TGFβ1 and those of TGFβ1 are antagonized by S100A9; TGFβ1 appears to pave the way for NT-S100A8-induced alterations in intracellular calcium.

### TGFβ1 and S100 protein interactions and their effects on cell proliferation, EMT and apoptosis

The proliferation of the four studied pancreatic cancer cell lines was not influenced by S100A8, S100A9, S100A8/A9 and/or TGFβ1 (Additional file 6: Table S1). Combined stimulation with NT-S100A8 and TGFβ1 induced an increase in MiaPaCa2 cell proliferation. We then verified whether proliferation was Smad4 sensitive by analyzing BxPC3 and BxPC3-SMAD4+ cell lines. As shown in Additional file 7: Figure S6, Smad4 expression was associated with a positive cell proliferation response to TGFβ1 and NT-S100A8, alone or combined, but not to the other molecules.

Since TGFβ1 is a strong promoter of the EMT, a critical step in carcinogenensis, we verified whether S100 calcium binding proteins are also involved in this process. To do this we first performed the relative quantification of Snai1, Snai2, Twist, Zeb1, Zeb2, CDH1 and CDH2 mRNA expression of two representative PDAC cell lines, BxPC3 and MiaPaCa2, with respect to the embryonic human cell line HEK293; the results are reported in Table 2.

**Table 2:** EMT genes mRNA relative quantification in the studied pancreatic cancer cell lines

	**BxPC3**	**MiaPaCa2**	**Hek293**
	**Fold increase**	**Fold increase**	**Reference**
**Snai1**	0.04	0.74	1
**Snai2**	0.11	2.06	1
**Twist**	0.48 × 10-3	0.38 × 10-3	1
**Zeb1**	0.12	0.32	1
**Zeb2**	0.87 × 10-3	Absent	1
**CDH1**	0.03	4.07 × 10-3	1
**CDH2**	0.04	Absent	1

**Figure 10:**
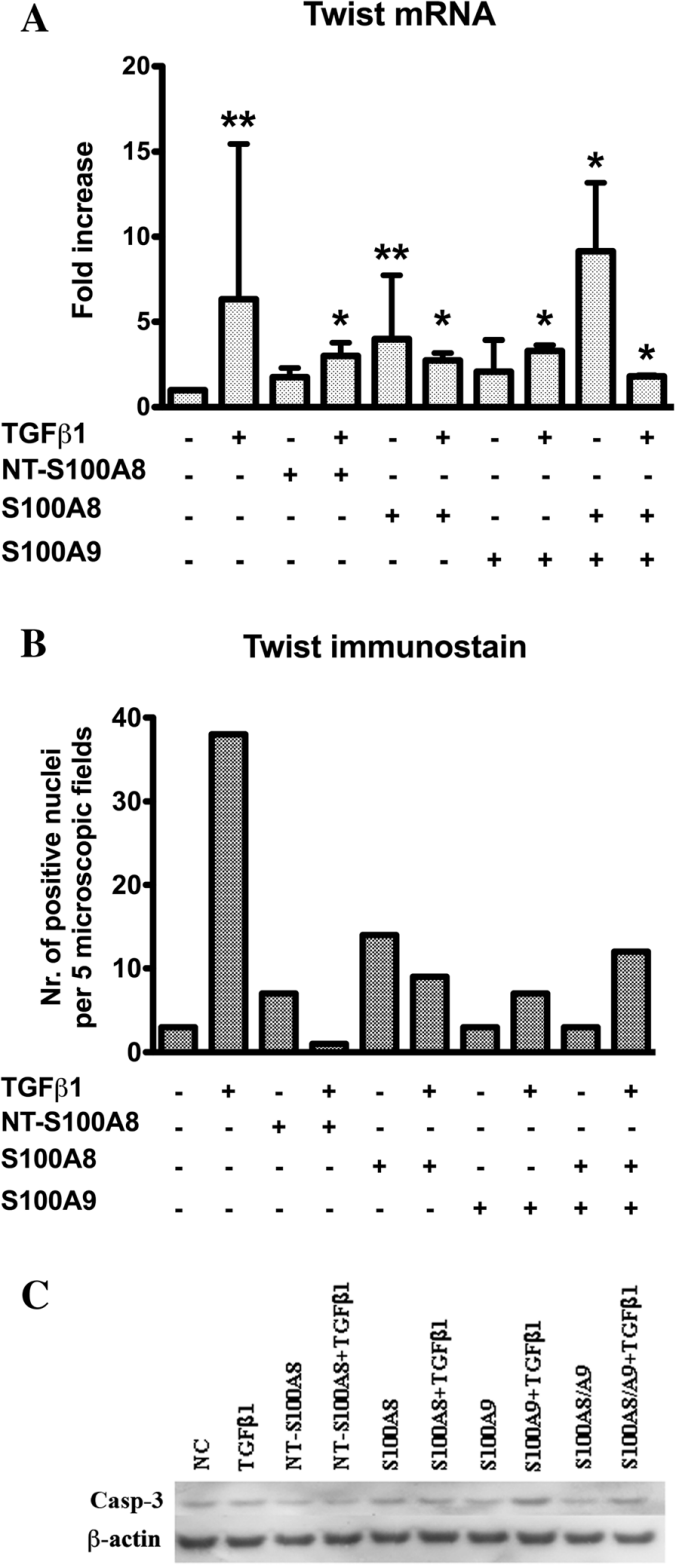
**EMT and apoptosis in BxPC3 cells. A:** Twist mRNA relative quantification of BxPC3 cells. Columns represent mean values, and bars represent standard deviations. Bonferroni’s test for pairwise comparisons: * = p < 0.05 with respect to non stimulated cells; ** = p < 0.01 with respect to non stimulated cells. **B:** Twist nuclear staining of BxPC3 cells. **C:** Western blot of cleaved caspase-3 (Casp-3) and β-actin, used as the control.

EMT gene expression levels were then ascertained in BxPC3 cells treated or untreated (control) with TGFβ1, S100A8, S100A9, and the S100A8/A9 complex and NT-S100A8. In BxPC3, TGFβ1 induced a 1.16 to 1.89 fold increase in all EMT genes except for Twist, which had a higher median fold increase (6.32 with respect to control cells). The relative expressions of Snai1, Snai2, Zeb1, Zeb2, CDH1 and CDH2 were not modified by S100 proteins, whereas Twist expression was significantly increased by S100A8 and by the S100A8/A9 complex to levels comparable with those of TGFβ1 (Kruskal-Wallis test: p = 0.018) (Figure 10A). The effects of TGFβ1, S100A8 and S100A8/A9 on Twist expression were much less evident when these molecules were simultaneously added to the cell cultures (Figure 10A). These findings were confirmed by those made at immunocytochemistry (Figure 10B). The E-cadherin protein was not expressed by untreated or treated BxPC3 cells. The findings for N-cadherin are shown in Additional file 8: Figure S7. Untreated cells showed dishomogeneous non-continuous membranous N-Cadherin immunostaining whereas TGFβ1, S100A8, S100A9 and NT-S100A8 immunostaining was less marked than in control cells. The S100A8/A9 complex caused enforced moderate to complete membranous immunostaining. When cells were co-stimulated with TGFβ1 and S100A8 or S100A9, weak complete immunostaining was observed. These data confirm that TGFβ1 is an EMT inducer and that Twist is its main target gene. Twist expression may also be enhanced by S100 calcium binding proteins but also by NT-S100A8. The effects of TGFβ1, S100A8 and S100A9 on EMT are mutually antagonistic when these molecules are added together.

To examine whether the studied molecules have pro-apoptotic effects on PDAC cells, cleaved caspase-3 was analyzed by western blot; this 17 kDa fragment was hardly detected (detectable only in fragments) in both control and treated Capan1, MiaPaCa2 and Panc1 cells. Cleaved caspase-3, present in BxPC3, was unaffected by any of the treatments (Figure 10C).

### TGFβ1 and S100 proteins interact and form heterocomplexes

**Figure 11:**
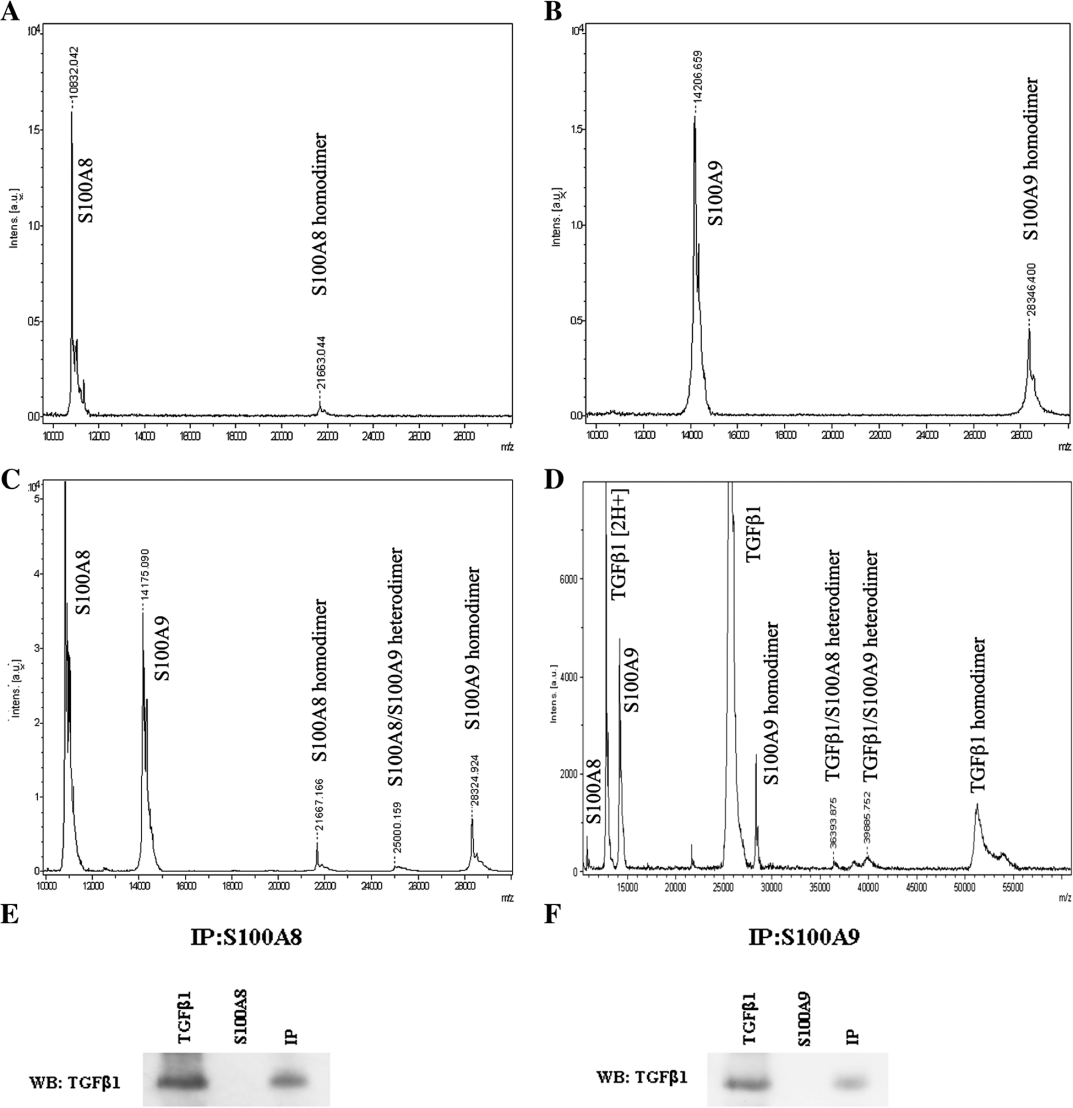
**S100A8, S100A9 and TGFβ1 molecular interactions.** S100A8, S100A9 and TGFβ1 and calcium ions (CaCl_2_) were mixed at an equimolar concentration (11.3 μM) in an aqueous solution, incubated overnight at 37°C and then submitted for MALDI-TOF/MS analysis. **A:** S100A8 alone; **B:** S100A9 alone; **C:** S100A8 and S100A9; **D:** S100A8, S100A9 and TGFβ1; **E:** TGFβ1 was incubated overnight at an equimolar concentration (1.6 μM) with S100A8 and CaCl_2_ at 37°C. Immunoprecipitation (IP) was performed using anti-S100A8. The immunoprecipitate underwent western blotting (WB) for the detection of TGFβ1. **F:** TGFβ1 was incubated overnight at an equimolar concentration with S100A9 and CaCl_2_ at 37°C. Immunoprecipitation (IP) was performed using anti-S100A9. The immunoprecipitate underwent western blotting (WB) for TGFβ1 detection.

In order to verify whether S100A8, S100A9 and TGFβ1 interact each other at a molecular level, these molecules were mixed at a 1:1 molar ratio in the presence of Ca^2+^, incubated overnight at room temperature and then analyzed by MALDI-TOF/MS. S100A8 formed homo-dimers, while S100A9 and TGFβ1 formed homo-dimers and homo-trimers. S100A8 and S100A9 formed hetero-dimers not only between themselves, but also with TGFβ1 (Figure 11). MALDI-TOF/MS findings were confirmed by co-immununoprecipitation experiments: TGFβ1 was detected in the fraction of polypeptides eluted from S100A8- and S100A9-Sepharose (Figure 11E and F, respectively). These data clearly indicate that TGFβ1, S100A8 and S100A9 are partner molecules.

## Discussion

The levels of calcium binding proteins S100A8 and S100A9 increase in cancer, including PDAC; although mainly expressed by tumor-infiltrating inflammatory cells, they may also be expressed by PDAC cells [31,34]. These proteins are directly involved in piloting metastases by the ligation of TLR4 and RAGE receptors [29,30,44]. The latter receptor, critical to bridging inflammation and cancer [45], and its ligation, initiates a feed-forward loop that potentiates inflammation, but may also favor the oncogenic switch [24,30]. In agreement with the hypothesis that S100A8 and S100A9 have a potential metastatic role, S100A8 and S100A9 mRNA levels were almost undetectable in the primary BxPC3, MiaPaCa2 and Panc1, while they were highly expressed by the metastatic SUIT2 and Capan1 PDAC cell lines [46]. Further supporting the potential role of S100A8/A9 proteins in metatases, high expression of their receptor, RAGE, was present not only in metastatic Capan1, but also in the primary MiaPaCa2 cells, which cause a liver metastases rate overlapping that of Capan1 [47]. The other S100A8/A9 receptor, TLR4 [29], is reported to activate STAT3 [48]. The finding that this signal transducer was constitutively activated in all PDAC cell lines, supports the hypothesis that it plays role in this cancer type [49]; however the fact that it was not induced by the S100 studied molecules, suggests that they act mainly through RAGE engagement.

In a previous study we demonstrated that PDAC tissue is enriched by the biologically active N-terminal 14 aa fragment of S100A8 (NT-S100A8) [34]. In this study, in order to further exploit tumor stoma interactions, we first verified whether tumor cells release soluble mediators able to fragment the S100A8 protein. Since the 12–15 aminoacid sequence IIDV of S100A8 fits with the group II consensus motif (L/IXX | XHy) of MMP2 [50], we ascertained whether MMPs have a role in causing the release of NT-S100A8. In the presence of PDAC -derived products, S100A8 resulted in a series of low molecular weight peptides, which were more abundant at 2280 m/z. At MS/MS analysis, the 2280 m/z peptide was found to correspond to the N-terminal 19 aa sequences of the original S100A8 protein, this being five aa longer than the PDAC-associated NT-S100A8 peptide [34]. The assumption that PDAC-derived MMPs might be responsible for S100A8 fragmentation and delivery of the 2280 m/z peptide was supported by the finding that S100A8 was not fragmented by conditioned media obtained from cells treated with Ukrain, which downregulates the activities of MMPs [41]. In the presence of PDAC cell conditioned media, the 2280 m/z peptide was resolved in two main degradation products at 1435 and 1030 m/z, which encompass the 8–19 and the 2–10 S100A8 aminoacid sequence, but also in a fragment at 1852 m/z, encompassing the 1–16 N-terminal aminoacid sequence of S100A8, which is very close to the NT-S100A8. This peptide was also detected when the entire S100A8 was incubated with Capan1 conditioned media, thus confirming that it derives from PDAC induced proteolytic cleavage of this calcium binding protein. Both BxPC3 and Capan1 conditioned media equally processed the 2280 m/z peptide, but the kinetics were different: metastatic Capan1 cells caused a more rapid (2 hours) degradation of the parental 2280 m/z peptide than the poorly metastatic BxPC3 cells (12 hours), and this indicates that PDAC cells, depending on their metastatic potential, produce different amounts of the same S100A8 targeting proteases. PDAC-associated S100A8 degradation may be governed by a regulatory mechanism restricting its function, but the release of peptides with their own biological activity might circumvent this phenomenon and modify tumor biology. We therefore studied the effects of S100A8, S100A9 and the S100A8/A9 complex, but also of NT-S100A8, on critical aspects of tumor biology, namely intracellular signaling, cell proliferation and EMT. On investigating NF-κB, Akt and mTOR signaling, we found that all these signaling pathways are differently targeted by S100A8, S100A9 and NT-S100A8 depending on the PDAC cell type (results summarized in Additional file 9: Figure S8). In PDAC cells with intact Smad4 (Capan1, MiaPaCa2 and Panc1), S100A8, S100A9 and S100A8/A9 shared an overall inhibitory effect on NF-κB, while these molecules did not affect NF-κB in the presence of SMAD4 homozygous deletion (BxPC3 cells). It has recently been suggested that NF-κB activation in PDAC is closely involved in driving tumor progression, especially when its activation is sustained, and that it plays a key role in linking inflammation and cancer [51]. Our findings suggest that S100 proteins, by targeting NF-κB signaling, may counteract cancer progression in Smad4 positive, but not in Smad4 negative, PDAC. However, other intracellular pathways, such as Akt, are critically involved in cancer proliferation, angiogenesis and metastases [43]. The two Akt activation sites, Thr^308^ and Ser^473^, were de-phosphorylated by S100A8, S100A9 and S100A8/A9 in Smad4 expressing cells, while Akt Thr^308^ was activated and Ser^473^ unaffected by all these molecules in Smad4 negative BxPC3 cells.

Overall, the above data suggest that Smad4 deletion is permissive for S100A8/A9 activation of NF-κB and Akt signaling pathways in PDAC cells. Interestingly, NT-S100A8 was far from ineffective and had effects similar to those of its parent molecule S100A8: the phosphorylation grade of the Akt Thr^308^ site was induced in BxPC3, and reduced in Capan1 and MiaPaCa2. In the PDAC setting, therefore, the degradation of S100A8 does not abolish its biological effects on PDAC cells, its NT-S100A8 proteolytic fragment maintaining them.

Akt signaling depends on a complex relationship with mTOR signaling: Akt activates mTOR complex 1 (mTORC1), while mTORC2 phosphorylate Akt on the hydrophobic motif Ser^473^[43]. However, in human PDAC, mTOR phosphorylation was more closely associated with cancer cells than Akt phosphorylation, which was found in the neoplastic ducts, but also in acinar, islet and stroma cells [52]. We therefore ascertained whether S100 proteins affected mTOR. As the catalytic subunit of the two distinct complexes, mTORC1 and mTORC2 [53], mTOR was sensitive to S100A8, which induced in BxPC3, Capan1 and MiaPaCa2 Ser^2481^ phosphorylation. Interestingly, the S100A9, but above all the S100A8/A9 complex, have opposite effects on mTOR: they induced mTOR phosphorylation in BxPC3, MiaPaCa2 and Panc1 and reduced it in Capan1. NT-S100A8 induced mTOR Ser^2481^, but also Ser^2448^ phosphorylation in almost all PDAC cell lines. Overall, our findings support the hypothesis that S100A8, S100A9 and NT-S100A8 are Janus-faced molecules [28] that can have pro- and anti-cancer effects, depending on the cellular context. Since the four cell lines studied differ in their differentiation grade but also have distinct genetic aberrations, Smad4 in particular [42,54], our hypothesis was that opposite responses to the same S100 molecule in different cell lines might depend on Smad4 status. To verify this, we selected cell without (BxPC3) and with (MiaPaCa2) Smad4 expression. BxPC3 were compared with BxPC3-SMAD4+ cells and MiaPaCa2 were compared with Smad4-siRNA-transfected MiaPaCa2 cells. Our findings confirmed that S100A8 does not inhibit or even activate NF-κB in the absence of Smad4 expression, but also supported the observation that S100A8 and NT-S100A8 activate mTOR independently from Smad4 status. Since Smad4 is critically involved in TGFβ1 signaling, it is reasonable to assume that there is a close link between these calcium binding proteins and TGFβ1 in pancreatic cancer. Therefore we also studied the combined effects of TGFβ1 and S100A8, S100A9, S100A8/A9 and NT-S100A8. TGFβ1 partly counteracted the effects of S100 proteins on NF-κB and Akt signaling in cells with Smad4 expression, but not those on mTOR. The effects of NT-S100A8 on Akt and mTOR, not those on NF-κB, were very similar to those exerted by S100A8, but were almost completely equal to those of TGFβ1 thus suggesting that NT-S100A8 might be functionally considered a TGFβ1-like molecule. The functional similarity between NT-S100A8 and TGFβ1 was also found when their effects on cell growth were evaluated. These two molecules synergistically stimulated the growth of MiaPaCa2 cells, but also the growth of BxPC3-SMAD4+, not that of BxPC3 cells, suggesting that this synergistic pro-proliferative effect is Smad4 dependent.

S100A8, S100A9, NT-S100A8 and TGFβ1 interactions were also found on intracellular calcium, which was analyzed because a crosstalk between calcium signaling and proliferation pathways has been well documented [35,37,38,55]. TGFβ1 accelerated the rapid train of intracellular calcium oscillations evoked by NT-S100A8 in BxPC3; this further supports the notion that these two molecules share effects and act in co-operation. Like NT-S100A8, the heterocomplex S100A8/A9, not any single molecule, evoked a sequence of intracellular calcium oscillations but, in the latter case, TGFβ1 counteracted the observed effect rather than reinforcing it. In MiaPaCa2, NT-S100A8 and S100A8 caused a reduction in intracellular calcium, and TGFβ1 had a similar effect. Importantly, S100A9 reversed the effects of TGFβ1, this finding further supporting the hypothesis that interactions may occur between TGFβ1 and S100 proteins.

In view of the interactions between S100 proteins and TGFβ1, and the fact that TGFβ1 is a well known inducer of EMT, a relevant biological phenomenon in morphogenesis and in cancer initiation and progression, we ascertained whether the observed molecular interactions also occur in this process. Of the two cell lines studied, the less differentiated and more aggressive MiaPaCa2 cell line expressed EMT genes at a level comparable to that of embryonic cells, suggesting a pre-existent EMT process. We therefore studied the more differentiated BxPC3 cells that, unlike the other cell lines, constitutively expressed cleaved caspase-3. However, none of the molecules studied induced this apoptotic marker. TGFβ1, as expected, induced an increased expression of all the EMT markers studied, while the S100 proteins induced only Twist mRNA expression, although in a different manner. The main inducers were the S100A8 and the S100A8/A9 complex, and these findings were confirmed at immunocytochemistry.

Since it has been claimed that Twist is involved not only in EMT, but also in inhibiting p53 mediated apoptosis, in the acquired resistance of cancer cells to chemotherapy and in circumventing oncogenic *k-ras* mediated cellular senescence [56,57], our results further indicate a link between the inflammatory S100 molecules and cancer progression. The effects of TGFβ1 and S100A8 on Twist expression were not cumulative, being reduced when both substances were added together, and S100A9, once again, counteracted the effects of TGFβ1. Interestingly, TGFβ1, but also S100A9 and NT-S100A8 reduced the membrane expression of N-Cadherin, while these effects were less pronounced when cells were co-stimulated with TGFβ1 and S100A9 or NT-S100A8.

All the above-described functional interactions may be due to different factors, including the sharing of co-receptors, and receptor interactions, but they might also be due to complex interactions between S100A8/A9 proteins and other molecules, like those occurring between S100A4 and amphiregulin [58] or S100B and basic fibroblast growth factor [59,60]. These interactions might prevent the canonical RAGE engagement by S100A8/A9 and enhance signaling of other receptors, like those of the interacting molecules, as demonstrated to occur when S100B interacts with βFGF [60]. We demonstrated by MALDI-TOF/MS and co-immunoprecipitation that TGFβ1 not only forms homo-dimers, but also homo-trimers and that this is true also for S100A9 (not shown), while S100A8 appears to almost exclusively form homo-dimers. It was confirmed that S100A8 and S100A9 form hetero-dimers but, as hypothesized, TGFβ1 was able to form hetero-complexes with both S100 molecules. *In vivo*, these interactions, occurring at the tumor stroma interface, probably contribute in the balance between the reported pro- and anti-tumor effects of S100A8/A9 in PDAC and further investigations of this phenomenon might allow the identification of new potential targets for intervention.

## Conclusions

In this study we demonstrated that in the PDAC setting important interactions occur between the calcium binding proteins S100A8 and S100A9 and TGFβ1. PDAC cells derived proteases target the inflammatory S100A8 protein causing the release of small peptides. One of these peptides, NT-S100A8, is biologically active and was demonstrated to exert TGFβ1-like effects on PDAC cell NF-κB, Akt and mTOR signaling and to synergize with TGFβ1 in altering intracellular calcium and in stimulating cell proliferation. Smad4 status affects S100A8 signaling, favoring mTORC1 when expressed and NF-κB when deleted. S100A9, by binding TGFβ1, counteracts the effects of this molecule on intracellular calcium, signaling and the EMT process.

## Materials and methods

### Cell lines

The pancreatic cancer cell lines used were: Panc1 and Suit2, donated by Prof Aldo Scarpa (University of Verona, Italy); BxPC3, from Dr Andrea Galli (University of Florence, Italy); MiaPaCa2, Capan1 and the human embryonic kidney Hek293 cell lines, supplied by the American Type Culture Collection (Manassas, VA, USA). BxPC3, Capan1 and Panc1 were cultured in RPMI (Gibco/BRL, Gaithhersburg, MD, USA) supplemented with 10% (BxPC3 and Panc1) and 20% (Capan1) fetal calf serum (FCS) (Gibco/BRL), 1% L-glutamine (Gibco/BRL) and 0.1% gentamycin (Gibco/BRL). MiaPaCa2 and Hek293 were cultured in DMEM (Gibco/BRL) supplemented with 10% FCS, 2% L-glutamine and 0.1% Gentamycin. BxPC3 cells were transfected with the expression vector pBK-cytomegalovirus (CMV)-SMAD4/DPC4, as previously described [34]. One SMAD4/DPC4-expressing clone (BxPC3-SMAD4+) was used for the experiments. Smad4 silencing of MiaPaCa2 cells was performed as previously described [61].

### Culture media for S100A8 proteolysis assay

To obtain BxPC3 and Capan1 conditioned media (CM), 400,000 cells were cultured (75 cm^2^ flasks) for 96 hours in RPMI supplemented with 1% FCS and 0.1% gentamycin. In parallel, Ukrain (Nowicky Pharma, Wien, Austria) treated Capan1 CM were obtained as above, the difference being that Ukrain was added 24 hours after cells had been seeded at the final concentration of 5 μM. Complete culture media maintained in a cell culture incubator for 96 hours (negative control), Capan1 and Ukrain-treated Capan1 CM were centrifuged at 1,500 rpm for 10 min, and then immediately mixed with 3 μM S100A8 or 2.5 μM S100A9 (ProSpec-Tany TechnoGene, Ness Ziona, Israel) and maintained at 37°C overnight before MALDI-TOF/MS analyses. Complete culture media, BxPC3 and Capan1 CM, treated as described above, were incubated at 37°C with 200 μM of the S100A8 1–19 N-terminal aminoacid sequence (NT-S100A8) (Primm, Milano, Italy) for 1, 2, 3, 4, 5, 6, 12 and 24 hours before MALDI-TOF/MS analyses. At least three independent sets of experiments were performed for both assays.

### Sample preparation for immunoblot analysis

Five million cells were seeded in Petri dishes (ø 10 cm) and cultured for 24 hours in their respective complete media prior to starting the experiments. Cells remained non-stimulated (negative control) or were stimulated with 50 mU insulin (positive control) (Insuman Rapid, Sanofi-Aventis, Deutschland, GmbH) for 10 min; in parallel, experimental cells were stimulated with 50 nM NT-S100A8, 0,02 ng/ml TGFβ1 (ProSpec-Tany TechnoGene), 10 nM S100A8 and 10 nM S100A9 alone or combined for 10 min. Following stimulations, Petri dishes were immediately transferred into an ice bath, and the cells were washed twice with cold PBS, re-suspended in 100 μl of cold lysis buffer [20 mM Tris–HCl, pH 7.5, 150 mM NaCl, 1 mM EDTA, 1% Triton-X 100, 50 mM NaF, 10 mM Na_4_P_2_O_7_,1 mM Na_3_VO_4_, and 10% protease inhibitor cocktail (Sigma Aldrich SRL, Milano, Italy)]. Lysates were centrifuged for 10 min at 14,000 rpm at 4°C and, in the supernatants, total proteins were measured using the Bio-Rad protein assay (Bio-Rad Laboratories, Milano, Italy). Each experiment was performed at least in triplicate.

### Immunoblot analysis and co-immunoprecipitation

For each sample, 40 μg proteins were electrophoresed through 4–12% NuPAGE®NovexBis-Tris SDS–PAGE Gel or 3-8% NuPAGE®NovexTris-Acetate SDS–PAGE Gel (Invitrogen S.R.L., Life Technologies, Monza, Italy) and electrophoretically transferred to Nitrocellulose Membrane (iBlot® Transfer Stack, Invitrogen S.R.L.) by means of the iBlotTMDry Blotting System (Invitrogen S.R.L.). Following incubation for 1 hour in a blocking buffer [5% low fat powder milk re-suspended in PBS-T (PBS with 0.1% Tween-20)], membranes were incubated overnight at 4°C with the primary antibodies [antiphospho IkB-α (Ser^32^) (Santa Cruz Biotechnology, Inc., Santa Cruz, CA, USA); anti-phospho-Akt (Ser^473^), anti-phospho-Akt (Thr^308^), anti-Akt and anti-phospho-S6 Ribosomal Protein (Ser^235/236^), anti-cleaved Caspase-3, anti-phospho-Stat3 Tyr^705^, anti-β-actin, anti-phospho mTOR (Ser^2448^, Ser^2481^) (Cell Signaling Technology, Danvers, MA, USA); anti-RAGE (R&D Systems, Minneapolis, MN, USA) ] diluted 1:5000 (β-actin), 1:2000 (mTOR, cleaved Caspase-3, Stat3) or 1:3000 (all the others) in the blocking buffer. The blots, washed three times in PBS-T for 15 min, were incubated with alkaline phosphatase-conjugated anti-rabbit (Cell Signaling Technology) or anti-goat (Sigma Aldrich) secondary antibodies and then washed three times in PBS-T for 15 min and developed with the ECL Advance Western Blot Detection Kit (GE Healthcare Technologies, Milan, Italy).

Co-immunoprecipitation experiments were performed using protein G-Sepharose (GE Healthcare Technologies). TGFβ1 (1.6 μM) was incubated in equimolar concentrations with S100A8 or S100A9 at 37°C overnight in the presence of equimolar CaCl_2_. The mixtures were then incubated for 2 h with anti-S100A8 polyclonal or anti-S100A9 monoclonal antibodies (Santa Cruz Biotechnology) preadsorbed to protein G. Immunoprecipitates, washed three times in 10 mM Tris HCl containing 40 mM NaCl, pH 7.5, were boiled in an SDS-PAGE sample buffer before electrophoresis on 4–12% NuPAGE®NovexBis-Tris SDS–PAGE gels. After protein transfer, nitrocellulose membranes were blotted with anti-TGFβ1 antibody (Cell Signaling Technology).

### Effects of S100A8, S100A9, NT-S100A8 and TGFβ1 on BxPC3 and MiaPaCa2 intracellular calcium ([Ca^2+^]_i_)

BxPC3 and MiaPaCa2 cells, seeded (80 × 10^3^ cells) on coverslips that had been inserted within each well of six well culture plates, were kept in their respective complete media for 24 hours. Coverslips were then incubated for 10 min at 37°C with 3 μM fluo-4 AM (Invitrogen S.R.L.) in an imaging buffer containing 137 mM NaCl, 5 mM KCl, 1.2 mM MgCl_2_, 0.44 mM KH_2_PO_4_, 4.2 mM NaHCO_3_, 5 mM glucose, and 20 mM HEPES pH 7.4. The cells were carefully washed with the imaging buffer after its removal. Calcium oscillations were then analyzed by means of an inverted epifluorescence microscope equipped with a top stage incubator (Tokai Hit INU-F1, Tokai Hit, Shizuoka-Ken, Japan) in order to keep cells in the optimal culturing environment. As an illumination source, we used a mercury arc discharge lamp and selected excitation and emission wavelengths with an FITC filter cube (EX 465–495 DM505 EM 515–555 nm). Use was made of an 60X1.45NA objective (Nikon Instruments S.P.A., Firenze, Italy) and, in order to minimize phototoxicity and photobleaching, a back-thinned electron multiplied CCD camera C9100-13 Imagem (Hamamatsu Photonics Italia S.R.L., Milano, Italy). After the first minute, required to achieve optimal culture conditions, intracellular fluorescence was continuously monitored (3 frames/sec) for 12 min. In all experiments, during the first minute of recording, the cells were maintained in imaging buffer for the first 2 minutes, after which they were stimulated with 10 nM S100A8, 10 nM S100A9 and 50 nM NT-S100A8, alone or in combination with 0.02 ng/ml TGFβ1. For all stimuli, additions were made directly to the microscope chamber without interrupting the recording. The fluorescence signal was quantified by measuring the mean pixel value of a manually selected cellular area for each frame of the image stack using the HCimage system (Hamamatsu Photonics Italia S.R.L.). For all the experimental conditions, at least 3 independents sets of experiments were performed.

### Real time PCR (RT-PCR) for EMT markers

BxPC3 and MiaPaCa2 cells (300 × 10^3^) were seeded in six well dishes for 24 hours in their complete media. Cells remained non-stimulated (negative control) or were stimulated with 10 nM S100A8, 10 nM S100A9 and 50 nM NT-S100A8, alone or combined with 0.02 ng/ml TGFβ1, for 72 hours. Following stimulation, the cells were collected with 0.25% trypsin (1 mM EDTA) and centrifuged for 10 min at 4,000 rpm. Total RNA was extracted from cells using the High Pure RNA isolation kit (Roche, Monza, Italy), and reverse transcription was performed using 200 ng of total RNA, M-MLV Transcriptase and 250 μM random primers (Invitrogen S.R.L.). Quantitative Real-Time PCR analysis for CDH1 (E-cadherin), CDH2 (N-cadherin), SNAI1 (Snail), SNAI2 (Slug), TWIST1 (Twist), ZEB1, ZEB2, and B2M (β2-micro-globulin) were performed with the LightCycler 480 Real-Time System (Roche Diagnostics, Mannheim, Germany). The primer sequences (shown in Additional file 10: Table S2) and the respective probes were designed using the Universal ProbeLibrary (Roche) and ProbeFinder software (http://www.roche-applied-science.com/). Experimental procedures followed the standard protocol using the LightCycler 480 Probes Master (Roche). β2-microglobulin (B2M) was included as a housekeeping gene control. At least three independent sets of experiments were performed in each experimental condition, and all real time PCR reactions were run in triplicate.

### Immunocytochemistry

In order to investigate immunocytochemical reactions, control and stimulated cells were formalin-fixed and paraffin embedded before obtaining 4–5 mm-thick sections from each cell block. All immunocytochemical staining was performed automatically (Bondt—maX, Menarini, Florence, Italy) with primary antibodies: E-cadherin (DakoCytomation, Glostrup, Denmark) (1:200), N-cadherin (DakoCytomation) (1:100), Twist1 (Novocastra, Newcastle-upon-Tyne, UK)(1:600). Sections were then lightly counterstained with hematoxylin. E-cadherin and N-cadherin immunoreactions were defined as immunoreactions detectable in the membrane while Twist was defined as a positive nuclear staining. At least three independent sets of experiments were performed in each experimental condition.

### MALDI-TOF/MS reflectron positive ion mode analysis

Cell culture media were first desalted and purified by ZipTip C18 pipette tips, following the procedure described in the ZipTip user’s guide. Five microliters of each purified peptide solution were added to 5 μl of α-cyano-4-hydroxycinnamic acid (HCCA) and 1 μl of the resulting mixture was deposited in duplicate on the steel sample holder and allowed to dry. MALDI-TOF/MS measurements were performed using an Ultraflex II MALDI-TOF instrument (BrukerDaltonics, Bremen, Germany), operating in reflectron positive ion mode. Ions were formed using a pulsed UV laser (λ = 337 nm) beam. The instrumental conditions were: IS1 = 25 kV; IS2 = 21.65 kV; reflectron potential = 26.3 kV; delay time = 0 nsec. External mass calibration (Peptide Calibration Standard, BrukerDaltonics) was based on monoisotopic values of [M + H] + of Angiotensin II, AngiotensinI, Substance P, Bombesin, ACTH clip (1–17), ACTH clip (18–39), Somatostatin 28 at m/z 1046.5420, 1296.6853, 1347.7361, 1619.8230, 2093.0868, 2465.1990 and 3147.4714, respectively.

### MALDI-TOF/MS linear positive ion mode analysis

S100A8, S100A9 and TGFβ1 and calcium ions (CaCl_2_) were mixed at an equimolar concentration (11.3 μM) in an aqueous solution. Each mixture was incubated overnight at 37°C prior to MALDI-TOF/MS analysis. S100A8, S100A9 and TGFβ1 mixtures were diluted 1/10 in 50 μL of 0.1% TFA aqueous solution, after which 5 microliters of each dilution were added to 5 μl of Sinapinic acid (SA); 1 μl of the resulting mixture was deposited in duplicate on the steel sample holder and allowed to dry. External mass calibration (Protein Calibration Standard II, BrukerDaltonics) was based on monoisotopic values of [M + H] + of Trypsinogen, Protein A, Albumin-Bovine (BSA), at m/z 23982, 44613, 66500, respectively. MALDI-TOF/MS measurements were performed using an Ultraflex II MALDI-TOF instrument (BrukerDaltonics), operating in linear positive ion mode. Ions were formed using a pulsed UV laser (λ = 337 nm) beam. The instrumental conditions were: IS1 = 25 kV; IS2 = 23.35 kV; delay time = 70 nsec. All chemicals and solvents were purchased from Sigma-Aldrich (Sigma Aldrich). One spectrum was collected for each sample, an average of 1,000 laser shots being obtained from the respective two replicate spots.

### Statistical analysis

The statistical analysis of data was made using one-way analysis of variance (ANOVA), Bonferroni’s test for pairwise comparisons, and Mann Whitney and Kruskal Wallis tests using the statistical software SPSS version 9.

## Abbreviations

RAGE: Receptor for advanced glycation endproducts; mTOR: Mammalian target of rapamycin; NF-κB: Nuclear factor kappa-light-chain-enhancer of activated B cells; MALDI-TOF/MS: Matrix-assisted laser desorption ionization- time-of-flight mass spectrometer.

## Competing interests

The authors declare they have no conflicting financial interests.

## Author’s contributions

Conception and design of the study: DB, DB, AP, SM. Generation, collection, assembly, analysis and/or interpretation of data: DB, DB, AP, SM, CFZ, PF, EG, MS, FS, FN, MF, MP, SD. Drafting and revision of the manuscript: DB, DB, AP, SM, AF. Approval of the final version of the manuscript: SP, MP. All authors read and approved the final manuscript.

## Supplementary Material

Additional file 1: Figure S1 Receptor for advanced glycation endproducts (RAGE) in PDAC cell lines. **A)** Western blot results obtained from PDAC cell lines (five millions in ø 10 cm Petri dishes) cultured for 24 hours in their respective complete media. Immunoblot was performed as described in Materials and Methods using Anti-RAGE (R&D Systems, USA) at a 1:200 dilution. **B)** Columns show the mean optical densities (O.D.; ImageJ software, v 1.47) of RAGE immunoblot results, obtained from 5 independent experiments. Bars show standard deviations. Click here for file

Additional file 2: Figure S2 MALDI-TOF-MS/MS sequencing of the 2280 m/z peptide. S100A8 was incubated with Capan1 conditioned media for 48 hours at 37°C before MALDI-TOF-MS/MS sequencing of the degration peptide at 1435 m/z. De novo sequencing was obtained by Mascot Distiller (v 2.5.0.0). Click here for file

Additional file 3: Figure S3 MALDI-TOF-MS/MS sequencing of the 1435 m/z peptide. S100A8 was incubated with Capan1 conditioned media for 48 hours at 37°C before MALDI-TOF-MS/MS sequencing of the degration peptide at 1435 m/z. De novo sequencing was obtained by Mascot Distiller (v 2.5.0.0). Click here for file

Additional file 4: Figure S4 Differential effects of S100 proteins and TGFβ1 on STAT signaling. BxPC3, Capan1, MiaPaCa2 and Panc1 remained unstimulated (NC, negative control) or were stimulated for 10 minutes with 50 mU insulin (positive control), 0.02 ng/ml TGFβ1 alone or combined with 50 nM NT-S100A8, 10 nM S100A8, 10 nM S100A9, 10 nM S100A8/A9 complex. Western blot shows Stat3 phosphorylation at Tyr^705^ site and the corresponding β-actin, used as control. Histograms show semi-quantification of band intensities after normalization against the negative control (O.D.; ImageJ software, v 1.47). Columns indicate mean values, bars indicate SD from two independ experiments. Click here for file

Additional file 5: Figure S5 Differential effects of S100 proteins and TGFβ1 on NF-κB, Akt, mTOR and STAT signaling in Smad4 expressing BxPC3 cells. BxPC3-SMAD4+ cells remained unstimulated (NC, negative control) or were stimulated for 10 minutes with 50 mU insulin (positive control), 0.02 ng/ml TGFβ1 alone or combined with 50 nM NT-S100A8, 10 nM S100A8, 10 nM S100A9, 10 nM S100A8/A9 complex. A: IκB-α phosphorylation at Ser^32^ site and corresponding β-actin. B: Akt phosphorylation sites Thr^308^ and Ser^473^ and corresponding non-phosphorylated Akt (panAKT). C: mTOR phosphorylation sites Ser^2481^ and Ser^2448^, phosphorylation of S6 Ribosomal Protein at the Ser^235/236^ site (pS6RP) and β-actin. D: Stat3 phosphorylation at Tyr^705^ site and corresponding β-actin. Histograms show semi-quantification of band intensities after normalization against the negative control (O.D.; ImageJ software, v 1.47). Columns indicate mean values, bars indicate SD from two independ experiments. Click here for file

Additional file 6: Table S1 XTT cell viability assay results. Click here for file

Additional file 7: Figure S6 XTT cell viability assay results. BxPC3 (empty columns) and BxPC3-SMAD4+ (filled columns) were seeded in a 96 well culture plate (2000 cells/well) and cultured for 48 hours, **A**: in absence (control) or in presence of TGFβ1 (0.02 ng/ml) and NT-S100A8 (50 nM), alone or combined; **B**: in absence (control) or in presence of TGFβ1 (0.02 ng/ml) and S100A8 (10 nM) and S100A9 (10 nM), alone or combined. In each experimental set, the median value of Abs_450nm_ of untreated cells was calculated and used as reference. All the other values were expressed as percentage to the reference. Columns and bars show mean and standard errors, respectivelty. Student’s t test: * = p<0.05. Click here for file

Additional file 8: Figure S7 N-cadherin immunostain. BxPC3 cells treated with NT-S100A8, S100A8, S100A9, S100A8/A9 alone (left panels) or combined (right panels) with TGFβ1. Click here for file

Additional file 9: Figure S8 Schematic representation of results. This study demonstrates that metastatic PDAC cells express S100A8/A9 mRNA transcripts and that both primary and metastatic PDAC-derived proteases cause the release of small peptides from the N-terminal sequence of S100A8. S100A8, S100A9 and S100A8/A9 heterocomplex share many effects on NF-κB, Akt and mTOR signaling in PDAC cells and these effects are dependent on SMAD4 expression (Smad4+=Smad4 expressing PDAC cells; Smad4- = Smad4 non expressing PDAC cells). The effects of the 14 aminoacid N-terminal sequence of S100A8 (NT-S100A8) may overlap those of the entire S100A8 or may differ. Continuous arrows indicate stimulation, dotted arrows indicate inhibition, dotted lines indicate no effect. TGFβ1 may counteract S100 proteins effects on NF-κB or Akt in SMAD4+, not in SMAD4-, PDAC cells. “Yes” indicates that TGFβ1 counteracts, “No” indicates that TGFβ1 does not counteract that specific effect of S100 proteins. *: only in Capan1 cell line. Click here for file

Additional file 10 **Supplementary Materials and Methods.** Table S2. Primers used for qRT–PCR analysis of the EMT genes. Click here for file
