# Supplementary figures and images for: Inflammation and pancreatic cancer: molecular and functional interactions between S100A8, S100A9, NT-S100A8 and TGFβ1
Source: Cell Commun Signal. 2014 Mar 26;12:20. doi: 10.1186/1478-811X-12-20 (PMC4108065; doi:10.1186/1478-811X-12-20)

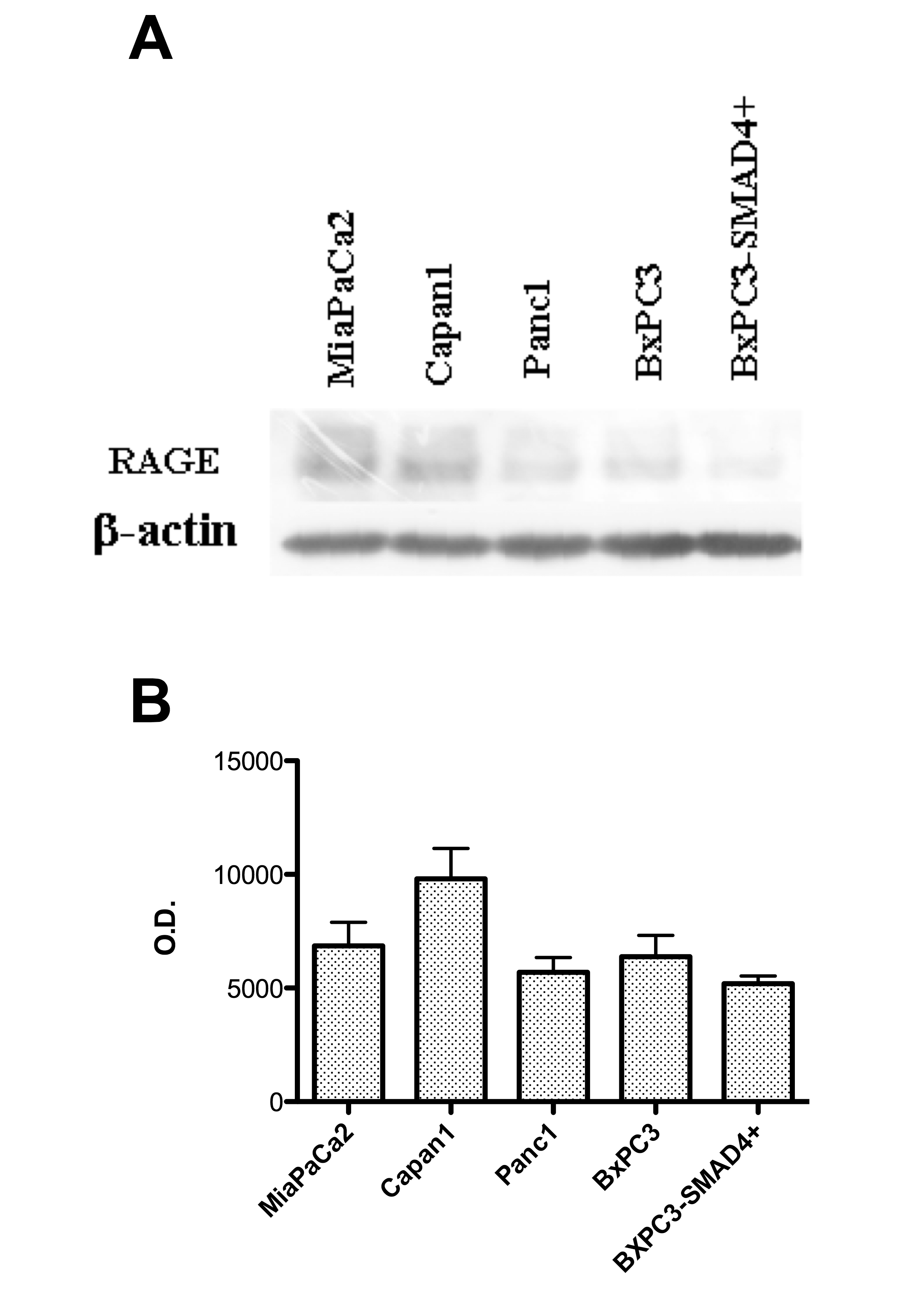

Supplement: Additional file 1: Figure S1 — Receptor for advanced glycation endproducts (RAGE) in PDAC cell lines. A) Western blot results obtained from PDAC cell lines (five millions in ø 10 cm Petri dishes) cultured for 24 hours in their respective complete media. Immunoblot was performed as described in Materials and Methods using Anti-RAGE (R&D Systems, USA) at a 1:200 dilution. B) Columns show the mean optical densities (O.D.; ImageJ software, v 1.47) of RAGE immunoblot results, obtained from 5 independent experiments. Bars show standard deviations. [file 1478-811X-12-20-S1.tiff]

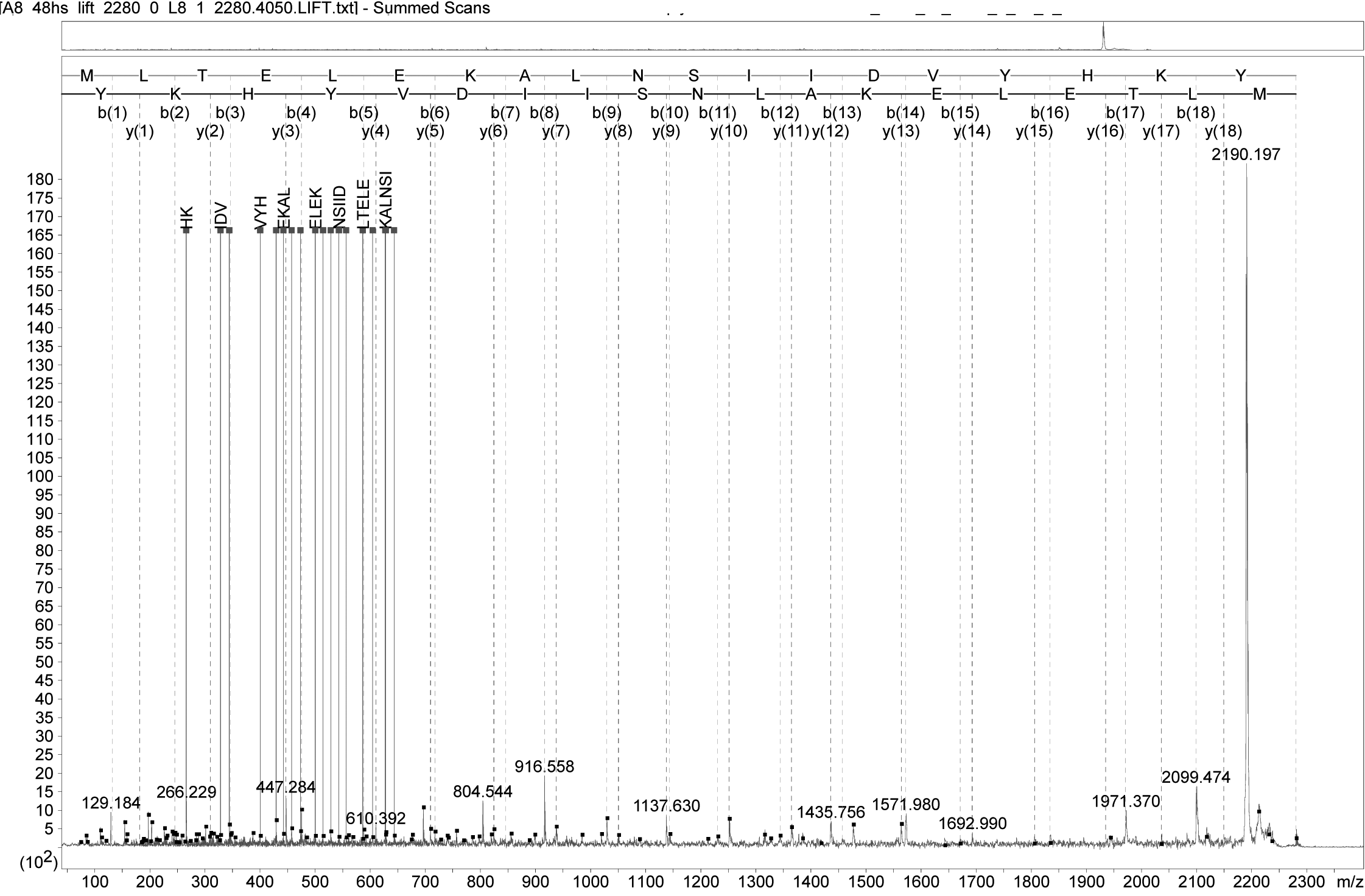

Supplement: Additional file 2: Figure S2 — MALDI-TOF-MS/MS sequencing of the 2280 m/z peptide. S100A8 was incubated with Capan1 conditioned media for 48 hours at 37°C before MALDI-TOF-MS/MS sequencing of the degration peptide at 1435 m/z. De novo sequencing was obtained by Mascot Distiller (v 2.5.0.0). [file 1478-811X-12-20-S2.tiff]

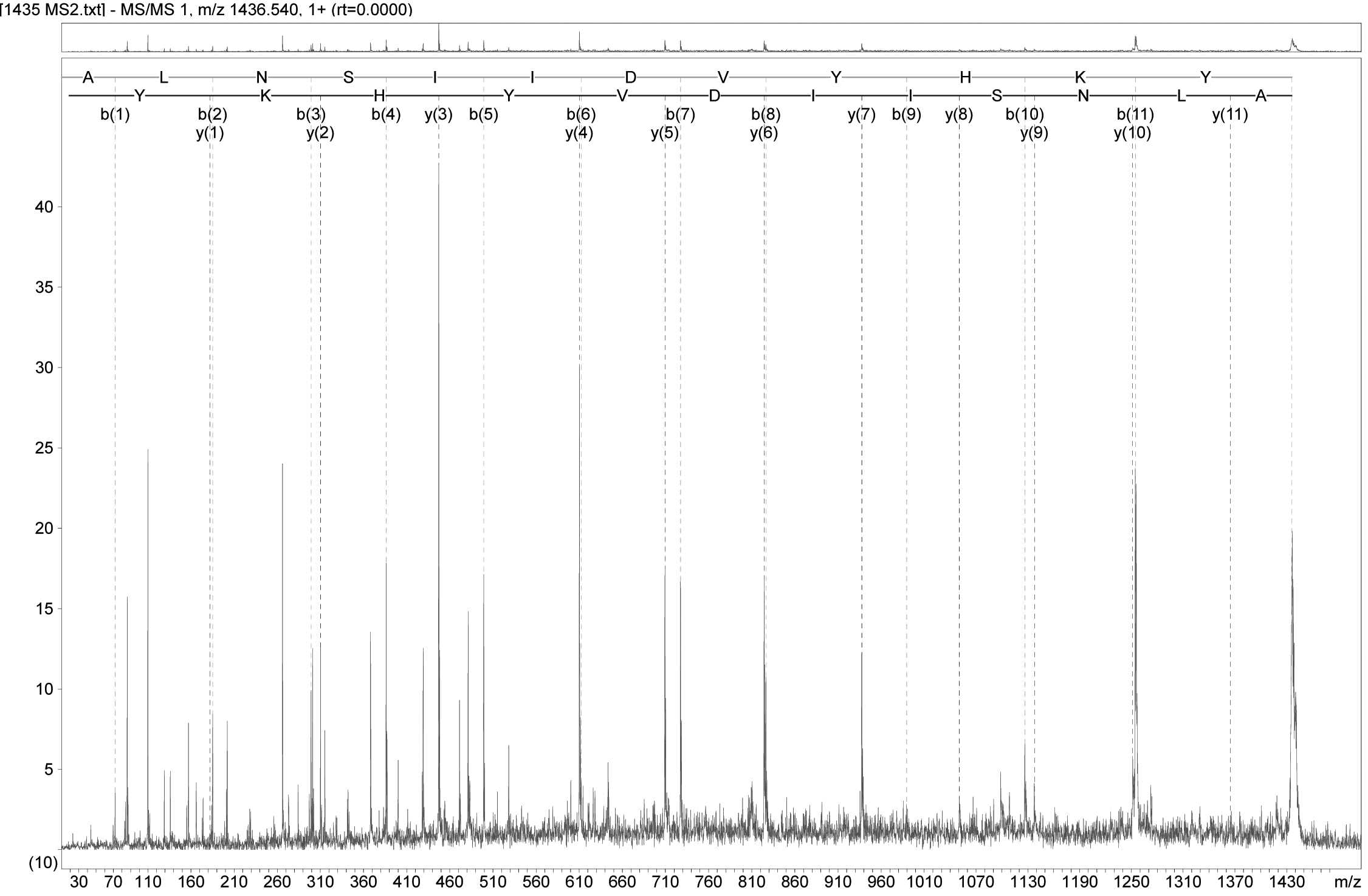

Supplement: Additional file 3: Figure S3 — MALDI-TOF-MS/MS sequencing of the 1435 m/z peptide. S100A8 was incubated with Capan1 conditioned media for 48 hours at 37°C before MALDI-TOF-MS/MS sequencing of the degration peptide at 1435 m/z. De novo sequencing was obtained by Mascot Distiller (v 2.5.0.0). [file 1478-811X-12-20-S3.tiff]

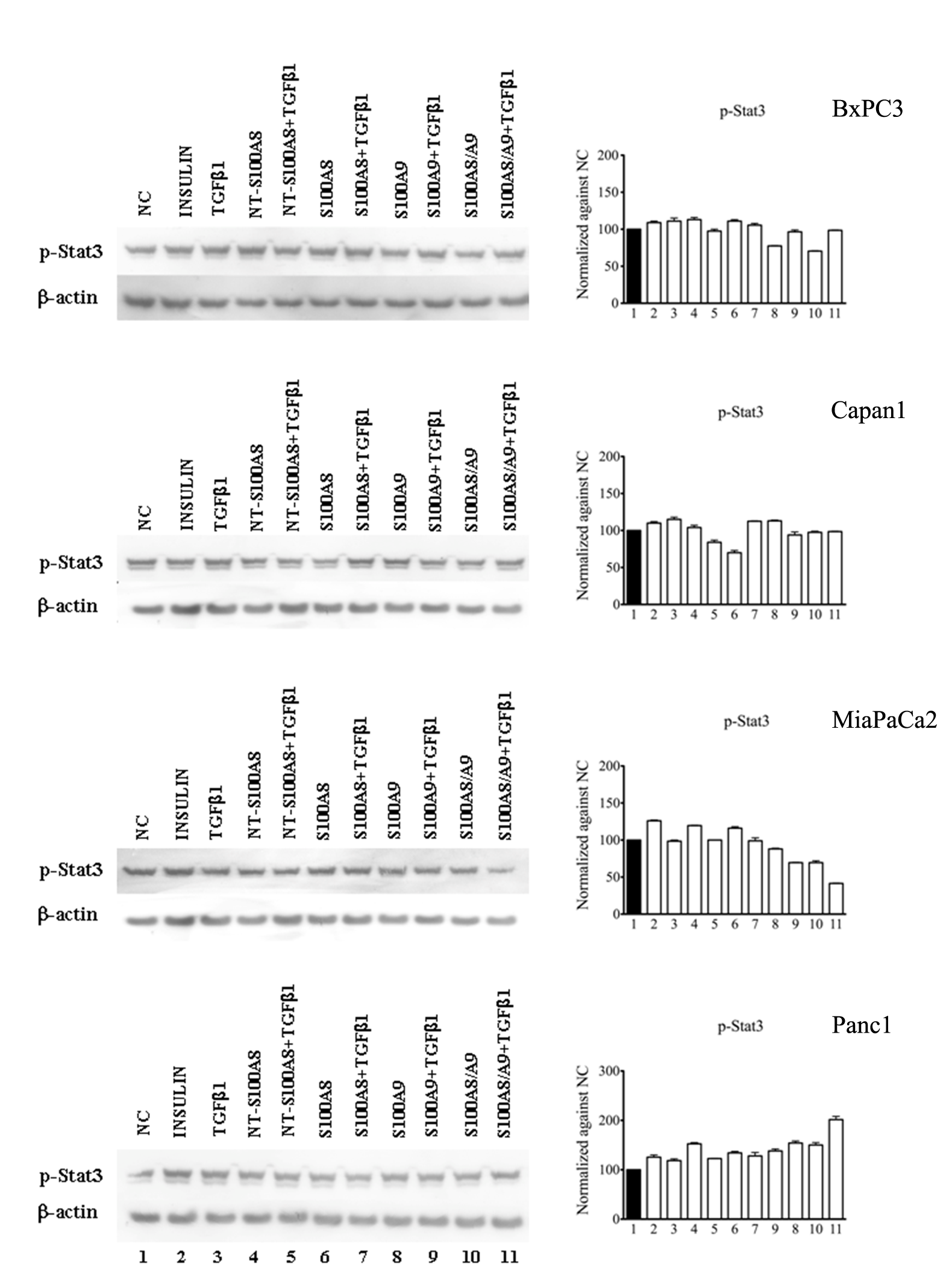

Supplement: Additional file 4: Figure S4 — Differential effects of S100 proteins and TGFβ1 on STAT signaling. BxPC3, Capan1, MiaPaCa2 and Panc1 remained unstimulated (NC, negative control) or were stimulated for 10 minutes with 50 mU insulin (positive control), 0.02 ng/ml TGFβ1 alone or combined with 50 nM NT-S100A8, 10 nM S100A8, 10 nM S100A9, 10 nM S100A8/A9 complex. Western blot shows Stat3 phosphorylation at Tyr705 site and the corresponding β-actin, used as control. Histograms show semi-quantification of band intensities after normalization against the negative control (O.D.; ImageJ software, v 1.47). Columns indicate mean values, bars indicate SD from two independ experiments. [file 1478-811X-12-20-S4.tiff]

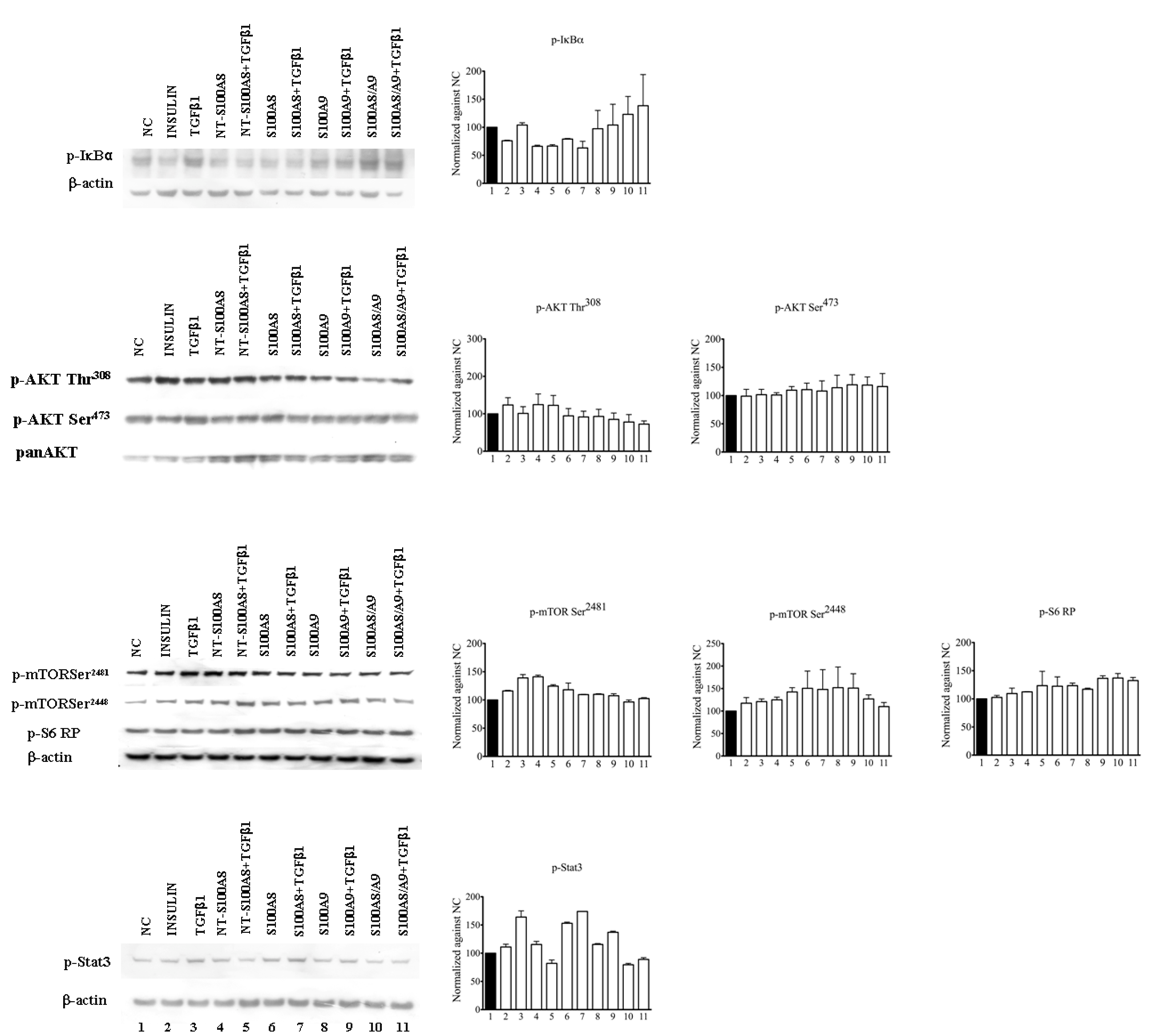

Supplement: Additional file 5: Figure S5 — Differential effects of S100 proteins and TGFβ1 on NF-κB, Akt, mTOR and STAT signaling in Smad4 expressing BxPC3 cells. BxPC3-SMAD4+ cells remained unstimulated (NC, negative control) or were stimulated for 10 minutes with 50 mU insulin (positive control), 0.02 ng/ml TGFβ1 alone or combined with 50 nM NT-S100A8, 10 nM S100A8, 10 nM S100A9, 10 nM S100A8/A9 complex. A: IκB-α phosphorylation at Ser32 site and corresponding β-actin. B: Akt phosphorylation sites Thr308 and Ser473 and corresponding non-phosphorylated Akt (panAKT). C: mTOR phosphorylation sites Ser2481 and Ser2448, phosphorylation of S6 Ribosomal Protein at the Ser235/236 site (pS6RP) and β-actin. D: Stat3 phosphorylation at Tyr705 site and corresponding β-actin. Histograms show semi-quantification of band intensities after normalization against the negative control (O.D.; ImageJ software, v 1.47). Columns indicate mean values, bars indicate SD from two independ experiments. [file 1478-811X-12-20-S5.tiff]

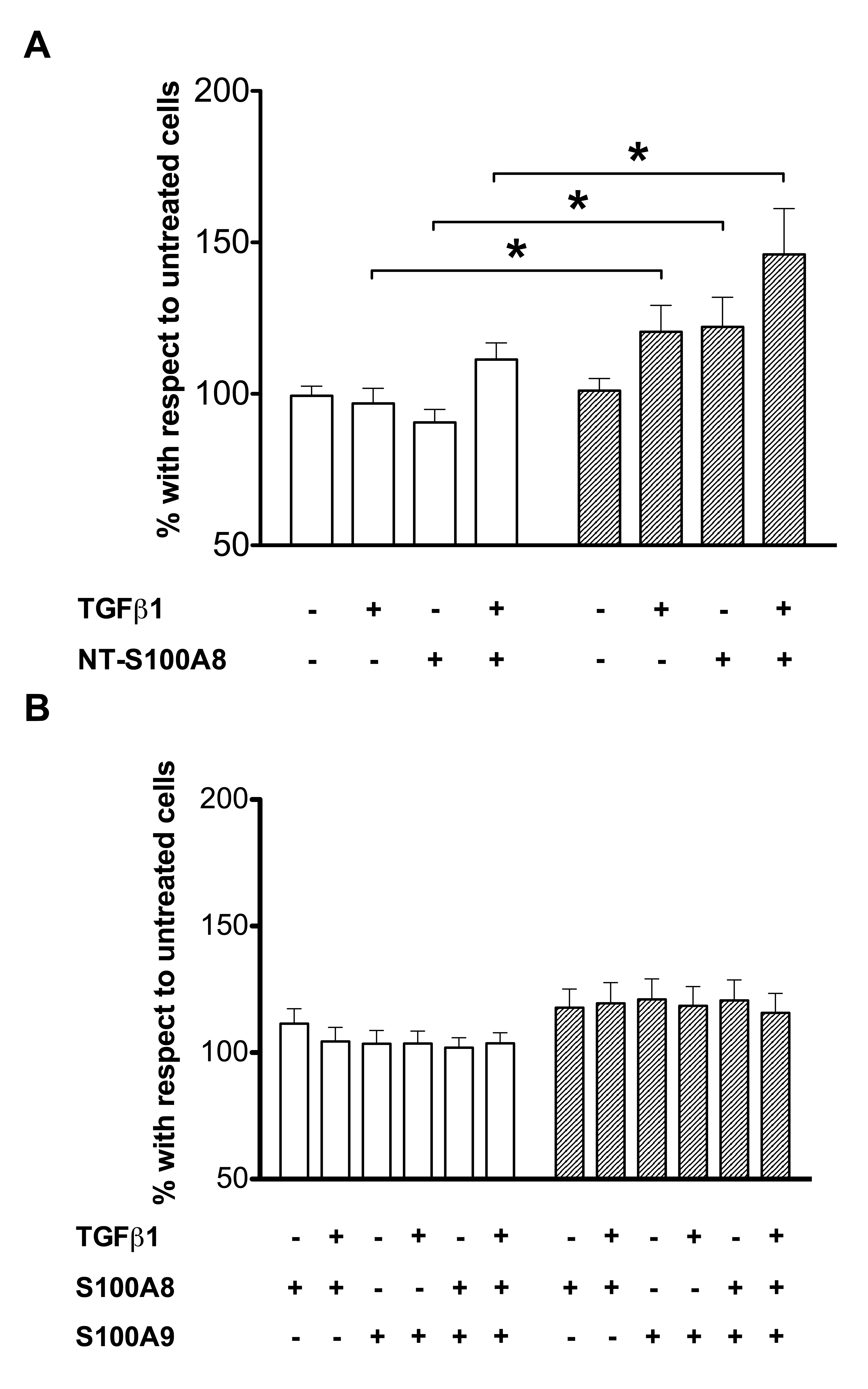

Supplement: Additional file 7: Figure S6 — XTT cell viability assay results. BxPC3 (empty columns) and BxPC3-SMAD4+ (filled columns) were seeded in a 96 well culture plate (2000 cells/well) and cultured for 48 hours, A: in absence (control) or in presence of TGFβ1 (0.02 ng/ml) and NT-S100A8 (50 nM), alone or combined; B: in absence (control) or in presence of TGFβ1 (0.02 ng/ml) and S100A8 (10 nM) and S100A9 (10 nM), alone or combined. In each experimental set, the median value of Abs450nm of untreated cells was calculated and used as reference. All the other values were expressed as percentage to the reference. Columns and bars show mean and standard errors, respectivelty. Student’s t test: * = p<0.05. [file 1478-811X-12-20-S7.tiff]

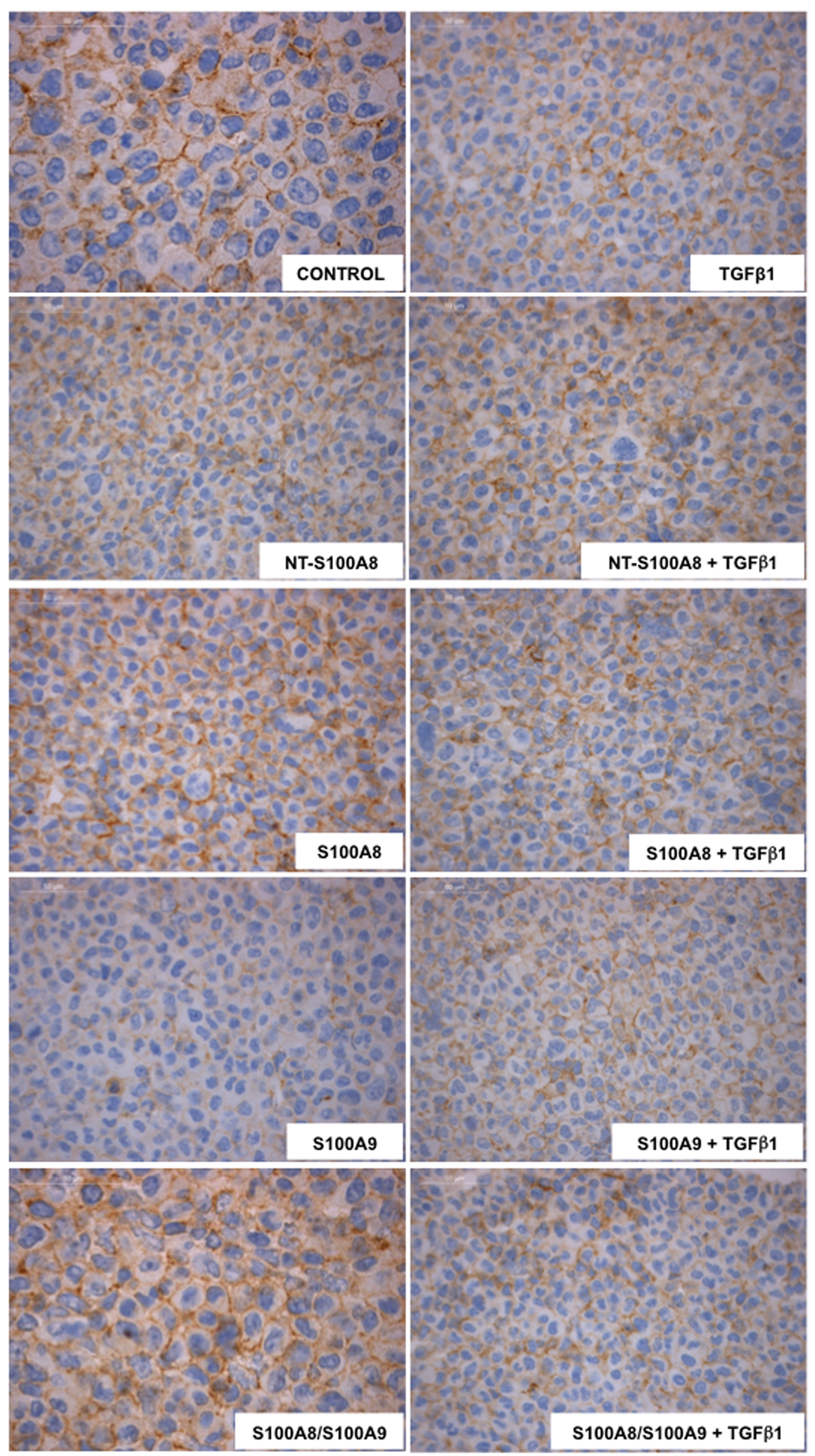

Supplement: Additional file 8: Figure S7 — N-cadherin immunostain. BxPC3 cells treated with NT-S100A8, S100A8, S100A9, S100A8/A9 alone (left panels) or combined (right panels) with TGFβ1. [file 1478-811X-12-20-S8.tiff]

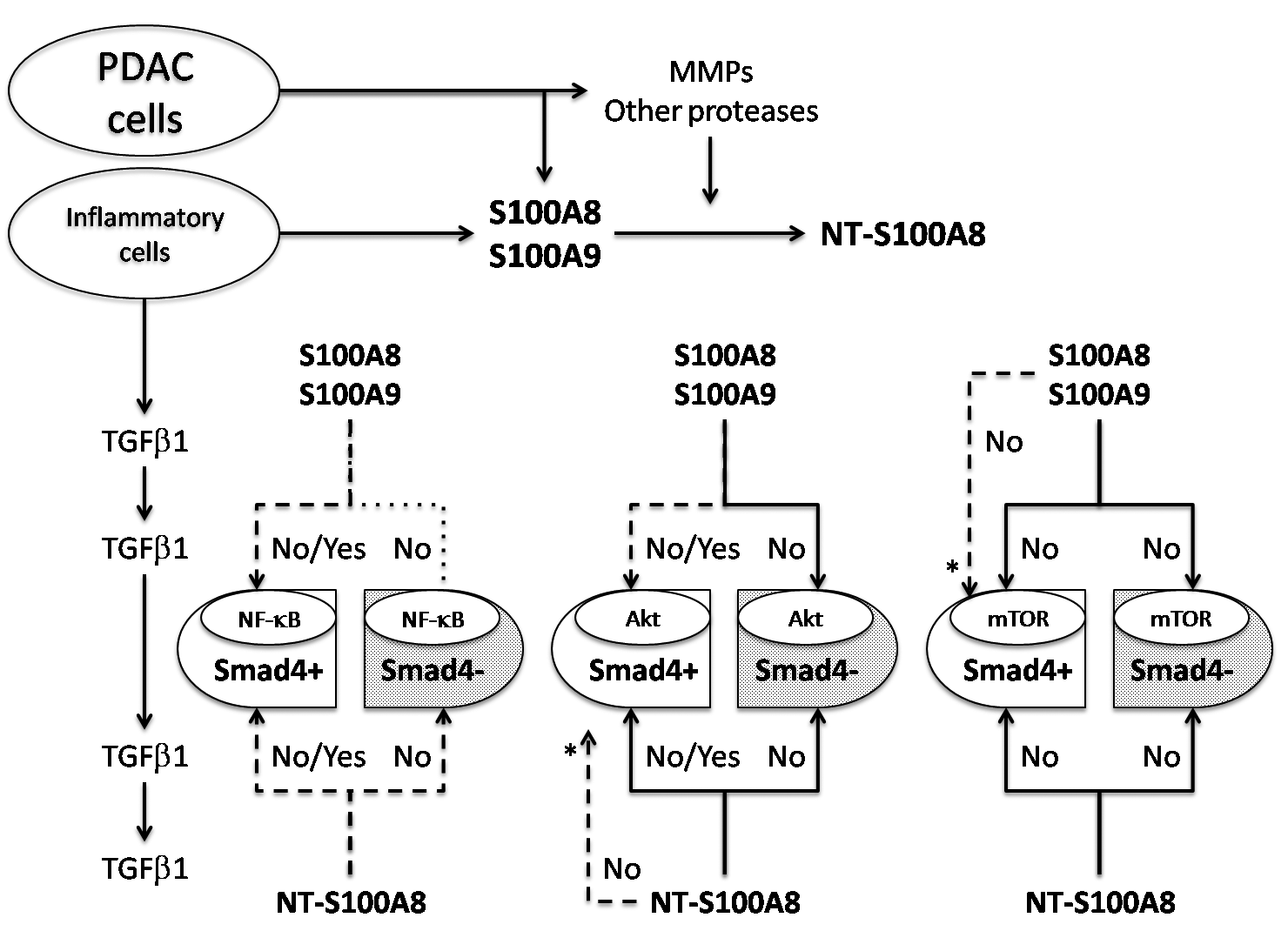

Supplement: Additional file 9: Figure S8 — Schematic representation of results. This study demonstrates that metastatic PDAC cells express S100A8/A9 mRNA transcripts and that both primary and metastatic PDAC-derived proteases cause the release of small peptides from the N-terminal sequence of S100A8. S100A8, S100A9 and S100A8/A9 heterocomplex share many effects on NF-κB, Akt and mTOR signaling in PDAC cells and these effects are dependent on SMAD4 expression (Smad4+=Smad4 expressing PDAC cells; Smad4- = Smad4 non expressing PDAC cells). The effects of the 14 aminoacid N-terminal sequence of S100A8 (NT-S100A8) may overlap those of the entire S100A8 or may differ. Continuous arrows indicate stimulation, dotted arrows indicate inhibition, dotted lines indicate no effect. TGFβ1 may counteract S100 proteins effects on NF-κB or Akt in SMAD4+, not in SMAD4-, PDAC cells. “Yes” indicates that TGFβ1 counteracts, “No” indicates that TGFβ1 does not counteract that specific effect of S100 proteins. *: only in Capan1 cell line. [file 1478-811X-12-20-S9.tiff]
