# Supplementary material for: Inflammation and pancreatic cancer: molecular and functional interactions between S100A8, S100A9, NT-S100A8 and TGFβ1
Source: Cell Commun Signal. 2014 Mar 26;12:20. doi: 10.1186/1478-811X-12-20 (PMC4108065; doi:10.1186/1478-811X-12-20)
Supplement: Additional file 6: Table S1 — XTT cell viability assay results. [file 1478-811X-12-20-S6.docx]

**Supplementary Table 1: XTT cell viability assay results.**

|  | BxPC3  % to Control  Mean±SEM | Panc1  % to Control  Mean± SEM | Capan1  % to Control  Mean± SEM | MiaPaCa2  % to Control  Mean± SEM |
| --- | --- | --- | --- | --- |
| Untreated cells (Control) | 99.38 ±3.18 | 98.27 ± 3.04 | 100.70 ± 2.18 | 99.47± 2.69 |
| TGFβ1 | 96.85 ± 5.02 | 88.31 ± 3.68 | 104.31 ± 2.68 | 102.11 ± 4.65 |
| NT-S100A8 | 90.58 ± 4.30 | 92.34 ± 3.44 | 105.44 ± 4.00 | 106.79 ± 4.27 |
| NT-S100A8 + TGFβ1 | 111.33 ± 5.54 | 91.40 ± 3.90 | 104.82± 3.24 | 128.07 ± 5.33 ** |
| S100A8 | 111.45±5.92 | 93.16 ± 3.65 | 96.22 ± 2.91 | 102.13± 4.93 |
| S100A8 + TGFβ1 | 104.44±5.53 | 85.25 ± 3.60 | 92.70 ± 1.99 * | 101.91 ± 4.46 |
| S100A9 | 103.53± 5.18 | 96.72± 4.43 | 103.07 ± 2.90 | 104.38± 4.09 |
| S100A9 + TGFβ1 | 103.55 ±4.99 | 89.11 ± 5.35 | 100.48 ± 2.27 | 97.35 ± 4.80 |
| S100A8/A9 | 101.96 ± 3.94 | 84.45 ± 4.50 | 101.72± 2.18 | 101.32 ± 4.40 |
| S100A8/A9 + TGFβ1 | 103.69 ± 4.17 | 85.81 ± 5.00 | 103.03 ± 2.44 | 105.42 ± 4.91 |
| One-way analysis of variance | F=1.651  p=0.102 | F=1.331  p=0.229 | F=2.153  p=0.031 | F=3.641  p<0.0001 |

Bonferroni’s test for pairwise comparisons:

*: p=0.06 vs NT-S100A8

**: p<0.05 vs all the other conditions
