# Supplementary material for: Inflammation and pancreatic cancer: molecular and functional interactions between S100A8, S100A9, NT-S100A8 and TGFβ1
Source: Cell Commun Signal. 2014 Mar 26;12:20. doi: 10.1186/1478-811X-12-20 (PMC4108065; doi:10.1186/1478-811X-12-20)
Supplement: Additional file 10 — Supplementary Materials and Methods. Table S2. Primers used for qRT–PCR analysis of the EMT genes. [file 1478-811X-12-20-S10.doc]

#### Supplementary Materials and Methods

#### Real time PCR (RT-PCR) for S100A8 and S100A9 mRNA quantification

Relative quantification of S100A8 mRNA was performed using RT-PCR protocol. Two micrograms of total RNA (High Pure RNA isolation kit, Roche, Monza, Italy) obtained from 1.0 × 10^6^ BxPC3, Capan1, MiaPaCa2, Panc1, BxPC3-SMAD4+ and Suit cell lines were reverse transcribed to cDNA using a random priming protocol (Random Primers, Invitrogen, San Giuliano Milanese, Italy) and the enzyme SuperScript™ II Reverse Trascriptase (Invitrogen). S100A8 and S100A9 mRNA were submitted for relative quantification by means of comparative C_T_ method. S100A8 and S100A9 were amplified starting with 500 ng cDNA. The primer pairs were:

5′CTCTTGTCAGCTGTCTTTCAGAAGA3′ (S100A8-F),

5′TTTCTCCAGCTCGGTCAACA3′ (S100A8-R),

5’CTCTTGTCAGCTGTCTTTCAGAAGA3’ (S100A9-F),

5’TTTCTCCAGCTCGGTCAACA3’ (S100A9-R).

TaqMan probes were:

5′6-FAM-AAGTCCGTGGGCATC-MGB3′ (S100A8-P) and

5’6-FAM-AAGTCCGTGGGCATC-MGB3’ (S100A9-P) (Applied Biosystems, Monza, Italy).

A concomitant quantification of HPRT1 mRNA (endogenous control) was performed for each experimental sample (PDARs part number 4326321E, Applied Biosystems) as described [34].

**XTT cell viability assay**

BxPC3, Capan1, MiaPaCa2, Panc1 and BxPC3-SMAD4+ cells were seeded (2000 cells per well) in 96-well cell culture plates and cultured in complete media for 24 hours. Media were then replaced with fresh cell culture media in absence (control) or in presence of TGFβ1 (0.02 ng/ml) and NT-S100A8 (50 nM), alone or combined or in absence (control) or in presence of TGFβ1 (0.02 ng/ml), S100A8 (10 nM) and S100A9 (10 nM) alone or combined. Cell growth was assessed after 48 hours using the XTT cell viability test (Roche, Milano, Italy). At least three independent experiements, each made in quadruplicate, were run. The results were expressed as percentage with respect to the median Abs_450nm_ of control cells run in the same experimental set.

**MALDI-TOF/MS/MS analysis**

MALDI-TOF-MS/MS sequencing of the 2280 and 1435 m/z peptides was performed on Applied Biosystems/MDS SCIEX 4800 MALDI TOF TOF instrument. S100A8 (3 μM) was incubated with Capan1 conditioned media for 48 hours at 37 °C before analysis. De novo sequencing was obtained by Mascot Distiller (v 2.5.0.0) selecting (b) and (y) ions.

**Supplementary Table 2.** **Primers used for qRT–PCR analysis of the EMT genes**.

**Gene Forward primer Reverse primer**

**CDH1** CCCGGGACAACGTTTATTAC GCTGGCTCAAGTCAAAGTCC

**CDH2** GGTGGAGGAGAAGAAGACCAG GGCATCAGGCTCCACAGT

**SNAI1** GCTGCAGGACTCTAATCCAGA ATCTCCGGAGGTGGGATG

**SNAI2** TGGTTGCTTCAAGGACACAT GTTGCAGTGAGGGCAAGAA

**TWIST1** CGGCCAGGTACATCGACT CATCTTGGAGTCCAGCTCGT

**ZEB1** GCCAACAGACCAGACAGTGTT TCTTGCCCTTCCTTTCCTG

**ZEB2** CAAGAGGCGCAAACAAGC AACCTGTGTCCACTACATTGTCA

**Internal Control**

**B2M** TTCTGGCCTGGAGGCTATC TCAGGAAATTTGACTTTCCATTC
